# Monocarboxylate Transporter-1 (MCT1)-Mediated Lactate Uptake Protects Pancreatic Adenocarcinoma Cells from Oxidative Stress during Glutamine Scarcity Thereby Promoting Resistance against Inhibitors of Glutamine Metabolism

**DOI:** 10.3390/antiox12101818

**Published:** 2023-09-30

**Authors:** Nourhane Ammar, Maya Hildebrandt, Claudia Geismann, Christian Röder, Timo Gemoll, Susanne Sebens, Ania Trauzold, Heiner Schäfer

**Affiliations:** 1 Institute of Experimental Cancer Research University Hospital Schleswig-Holstein, Campus Kiel, Arnold-Heller-Str. 3, Bldg. U30, 24105 Kiel, Germany; nourhane.h.ammar@gmail.com (N.A.); maya.hildebrandt@gmx.de (M.H.); susanne.sebens@email.uni-kiel.de (S.S.); atrauzold@email.uni-kiel.de (A.T.); 2 Department of Internal Medicine and Gastroenterology, Carl-von-Ossietzky University Oldenburg, Philosophenweg 36, 26121 Oldenburg, Germany; claudia.geismann@uni-oldenburg.de; 3 TriBanK, University Hospital Schleswig-Holstein, Campus Kiel, Arnold-Heller-Str. 3, Bldg. U30, 24105 Kiel, Germany; christian.roeder@uksh.de; 4 Section for Translational Surgical Oncology & Biobanking, Department of Surgery, University of Lübeck, Ratzeburger Allee 160, 23562 Lübeck, Germany; timo.gemoll@uksh.de

**Keywords:** tumor metabolism, anaplerosis, drug resistance, pancreas

## Abstract

Metabolic compartmentalization of stroma-rich tumors, like pancreatic ductal adenocarcinoma (PDAC), greatly contributes to malignancy. This involves cancer cells importing lactate from the microenvironment (reverse Warburg cells) through monocarboxylate transporter-1 (MCT1) along with substantial phenotype alterations. Here, we report that the reverse Warburg phenotype of PDAC cells compensated for the shortage of glutamine as an essential metabolite for redox homeostasis. Thus, oxidative stress caused by glutamine depletion led to an Nrf2-dependent induction of MCT1 expression in pancreatic T3M4 and A818-6 cells. Moreover, greater MCT1 expression was detected in glutamine-scarce regions within tumor tissues from PDAC patients. MCT1-driven lactate uptake supported the neutralization of reactive oxygen species excessively produced under glutamine shortage and the resulting drop in glutathione levels that were restored by the imported lactate. Consequently, PDAC cells showed greater survival and growth under glutamine depletion when utilizing lactate through MCT1. Likewise, the glutamine uptake inhibitor V9302 and glutaminase-1 inhibitor CB839 induced oxidative stress in PDAC cells, along with cell death and cell cycle arrest that were again compensated by MCT1 upregulation and forced lactate uptake. Our findings show a novel mechanism by which PDAC cells adapt their metabolism to glutamine scarcity and by which they develop resistance against anticancer treatments based on glutamine uptake/metabolism inhibition.

## 1. Introduction

Alterations in tumor cell metabolism together with changes in the metabolic microenvironment essentially contribute to tumor heterogeneity [1,2,3]. Manifesting in distinct compartments of the release or consumption of certain metabolites such as lactate, this heterogeneity results in a metabolic flux that variably provides the cancer cells with energy, biomass, redox mediators, and epigenetic modulators [4,5,6]. The vast majority of cancer cells utilize high amounts of glucose through glycolysis under hypoxic conditions (anaerobe glycolysis) as well as normoxic conditions (aerobe glycolysis or Warburg metabolism). In contrast to normal cells, these tumor cells running glycolysis under normoxia metabolize only a small fraction of the glycolytic end-product pyruvate through oxidative phosphorylation to yield energy. Instead, pyruvate is mainly reduced to lactate, which is then released out of the cell [7,8]. In this way, the maintenance of glycolysis by the recovery of NAD+ from pyruvate reduction is ensured, while the energy demand of the cells is largely covered by excessive glycolysis (yielding two ATP per one glucose). This high rate of glycolysis in Warburg cells feeds biosynthetic pathways for nucleotide synthesis and biomass production, in line with a highly proliferative phenotype [8].

Interestingly, it has been shown that these Warburg cells depend more on the availability of NAD+ than on the generation of ATP [9]. To supplement other metabolic pathways such as the citric acid cycle (CAC) and those favoring a forced growth rate, highly glycolytic cancer cells require additional metabolites. Amongst these metabolites, glutamine plays an essential role [10,11,12] and most tumors, including pancreatic cancer [13,14], therefore depend on both glucose and glutamine [15,16]. Glutamine, either directly or after its conversion to glutamate, feeds the exchange systems for other amino acids as well as the citric acid cycle (anaplerosis), thereby serving as a nitrogen source as well as a substrate for biomass synthesis. Moreover, glutamine essentially contributes to redox homeostasis [17,18] through the glutathione antioxidant system. Given this pivotal role in tumor cell metabolism, novel therapeutic strategies are envisioned [15,19] aiming to target the uptake or conversion of glutamine [20,21,22] and thereby to suppress the growth of cancer cells addicted to it [23].

Besides high-rate glycolysis, some cancer cells utilize glucose in a rather oxidative fashion while others even use lactate as a substrate for oxidative metabolism [24,25]. Particularly the presence of the latter type of cancer cells has been reported to be associated with greater malignancy of many tumors, including melanoma, glioblastoma, and multiple myeloma, as well as pancreatic, colorectal, ovarian, prostate, bladder, head–neck, breast, and lung cancer [24,25,26,27,28,29,30,31,32,33,34,35]. These cells have been termed reverse Warburg cells as they metabolize the lactate secreted by Warburg cells [36,37,38]. In many tumors, the latter not only consist of cancer cells but also stromal cells such as cancer-associated fibroblasts that run high-rate glycolysis and secrete lactate [26,38,39]. In stroma-rich cancers like pancreatic ductal adenocarcinoma (PDAC), stromal and tumor cells create glycolytic lactate-producing (Warburg) and oxidatively lactate-consuming (reverse Warburg) compartments. The flux of lactate between the cells of either compartment is driven by members of the SLC16 protein family. Lactate secretion by the Warburg cells mainly occurs through the lactate carrier SLC16A3, also termed monocarboxylate transporter 4 (MCT4), whereas the reverse Warburg cells import lactate through SLC16A1, also termed monocarboxylate transporter 1 (MCT1). Thus, reciprocal MCT1/MCT4 expression patterns in different tumor areas could reflect the lactate exchange between the Warburg and reverse Warburg cell compartments [40]. The latter compartment has recently been shown to give rise to more aggressive and metastatic tumors [41,42]. This has been reported for malignant melanoma [35] or HER2+ breast cancer [43] in which MCT1-mediated lactate import favors cancer cell dissemination and metastatic growth. Moreover, the reverse Warburg compartment in PDAC, triple-negative breast cancer, and glioblastoma multiforme provides a niche harboring cancer stem cells (CSCs) that are maintained through lactate uptake through MCT1 while experiencing glucose starvation [32,44,45].

Besides its role as a substrate for oxidative energy production, lactate taken up from the environment also serves as a metabolite that modulates the cellular redox balance [46,47,48] and could lead to epigenetic alterations [49,50]. Given the broad range of its effects on cellular processes in cancer cells and the resulting phenotypical changes depending on lactate utilization, its blockade by MCT1 inhibitors such as AZD3965 is currently in clinical testing [51]. Since the metabolic coupling between Warburg and reverse Warburg cells may not be limited to the different utilization of glucose and lactate, the aim of this study was to investigate whether MCT1-driven lactate import in PDAC cells is connected to glutamine metabolism and whether lactate utilization compensates for glutamine depletion and confers resistance against glutamine uptake inhibitors.

## 2. Materials and Methods

### 2.1. Cell Lines and Culture

Experiments were carried out on the human pancreatic ductal adenocarcinoma (PDAC) cell lines T3M4 (kindly provided by H. Friess, Heidelberg, Germany) and A818-6 (a kind gift from H. Kalthoff, Kiel, Germany). Culture conditions were performed as described previously [52,53]. Cell line authenticity was checked by STR-profiling.

### 2.2. RNA Preparation and Real-Time PCR

Isolation of RNA was performed using the Monarach^®^ Total RNA Miniprep Kit (NEB, Frankfurt, Germany) following the manufacturer’s instructions. Reverse transcriptions into single-stranded cDNA and qPCR (Real-Time Thermocycler CFX Connect™, Bio-Rad, Feldkirchen, Germany) were carried out using the SYBR-Green assay (Blue S’ Green qPCR, Biozym, Hamburg, Germany). All primers (Eurofins, Ebensburg, Germany) were used at a final concentration of 0.2 µM. Cycling conditions were as follows: 95 °C/3 min, 95 °C/10 sec for denaturation, 57 °C/20 s for annealing of GCLC, 60 °C/20 s for MCT1, MCT4 and RPL13, and 78 °C/10 s for extension. Data analysis was performed using the Bio-Rad CFX Maestro software. The following primer sets were used: RPL13: forward 5′-cctggaggagaagaggaaagaga-3′/reversed 5′-ttgaggacctctgtgtatttgtcaa-3′ (NM_012423.4); MCT1: forward 5′-tccagctctgaccatgattg-3′/reversed 5′-gcccccaagaattagaaagc-3′ (NM_003051.4); MCT4: forward 5′-ctcgtggtcttctgcatctt-3′/reversed 5′-aaaatcagggaggaggtgag-3′ (NM_001206950.2); GCLC: forward 5′-agttgaggccaacatgcgaa-3′/reversed 5′-tgagcgagggtgcttgttt-3′ (NM_001498.4).

### 2.3. Western Blotting

Total cell lysates were prepared using 2xLaemmli buffer, separated with SDS-PAGE for Western blot analysis, as described before [53,54]. The target proteins were visualized and analyzed using the ChemiDoc™ MP Imaging System (Bio-Rad). The density of the bands was estimated using Image Lab™ software 6.1 (Bio-Rad). The relative expression of investigated proteins was calculated by normalizing the band intensities of the target protein to those of the housekeeping protein HSP90. MCT1, MCT4, Lamin A/C, and GCLC antibodies (all from Santa Cruz, Heidelberg, Germany) were 1:200 diluted in 5% skimmed milk in TBS-Tween (TBST); HSP90 and Nrf2 antibodies (Cell Signaling, Frankfurt, Germany) were 1:1000 and 1:400 diluted, respectively, in 5% BSA in TBST.

### 2.4. siRNA Transfection

For siRNA transfection, cells grown in 12 well-plates were transfected with 20 µM of control siRNA (AllStars Negative Control siRNA, Qiagen GmbH, Hilden, Germany), MCT1 (S103246614, Qiagen), or Nrf2 siRNA (S103246614, Qiagen), using 6 µL HiperFect transfection reagent (Qiagen) and 100 µL Opti-MeM^®^ (Gibco 31985-047).

### 2.5. MCT1 Surface Immunostaining and Flow Cytometry

Cells were collected, washed, and resuspended in MACS buffer (PBS with 5 mM EDTA, 2% (*w*/*v*) BSA). Then, cells were stained with an MCT1-antibody recognizing the N-terminus (Santa Cruz) at 1:200 in MACS buffer or a mouse IgG control antibody (Santa Cruz) for 1 h at room temperature. After extensive washing, cells were incubated with donkey antimouse Alexa Fluor^®^-647 (Biolegend, Amsterdam, The Netherlands) at 1:500 dilution for 1 h at room temperature, followed by one wash step and fixation in 1% (*v*/*v*) formaldehyde in MACS buffer. Immunostaining was analyzed by fluorescence flow cytometry on a FACSVerseTM instrument (Becton Dickinson, East Rutherford, NJ, USA) using FACSuite Flow Cytometry Software (Becton Dickinson).

### 2.6. Lactate Uptake Assay

T3M4 and A818-6 cells (1–2 × 10^4^/well) grown in 12-well plates were equilibrated in a glucose-free medium. Then, the medium was replaced by 10 mM HEPES/pH 7.50, 5 mM KCl, 100 mM NaCl, and 1 mM MgCl_2_ (uptake buffer) containing 2 μCi (0.5 μM) ^14^C-L-lactate (American Radiolabeled Chemicals, Inc. ARC 0593, Saint Louis, MO, USA) alone (=total) or together with 10 mM unlabeled lactate (to detect unspecific binding). Cells were incubated for 1 to 3 h at 37 °C. Afterwards, cells were washed 3× with ice-cold phosphate-buffered saline and then lysed in 500 μL uptake buffer with 2% (*w*/*v*) sodium dodecyl sulfate. Lysates were submitted to liquid scintillation beta counting (LS-6500 instrument, Beckman Coulter, Krefeld, Germany). In parallel, protein concentrations in lysates from unlabeled cells were measured (DC Assay, BioRad) and used for the normalization of beta counting rates (triplicate measurements). Specific C14-lactate uptake was calculated by subtracting the normalized counting rates (total minus unspecific).

### 2.7. Propidium Iodide Staining

To study cell cycle progression and apoptotic cell death indicated by the subG1 fraction, cells were trypsinized and washed twice in cold PBS containing 5 mM EDTA (PBS/EDTA). Afterwards, they were resuspended in 500 µL PBS/EDTA and fixed by adding 1 mL chilled EtOH dropwise followed by incubation on ice for 20 min. Fixed cells were then collected by centrifugation, resuspended in 200 µL PBS/EDTA, and incubated with RNaseA for 30 min at room temperature to ensure staining of only DNA. Then, cells were subsequently stained with propidium iodide (PI) by adding 200 µL of a 200 mg/mL PI-stock solution (Sigma^®^, Taufkirchen, Germany). Samples were stored at 4 °C in the dark until counting using BD FACSVerse cytometer (Becton Dickinson).

### 2.8. Glutathione (GSH) Colorimetric Assay

For quantitative analysis of cellular GSH content, a colorimetric 96-well microplate assay from the Zellx^®^ assay kit (Zellbio, Lonsee, Germany) was used according to the manufacturer’s instructions. Cells grown in 6-well plates were washed twice with prechilled PBS and kept on ice until lysis. To calculate the volume of lysis buffer to be used, the number of cells was counted from those cultured and treated in parallel. Then, 1 mL of lysis buffer (5% *w*/*v* sulfosalicylic acid (SSA) in H_2_O) per 2 × 10^6^ cells was added and the plates were frozen at −80 °C for at least 16 h. After thawing on ice, lysates were scraped off the plates, vigorously vortexed, and placed on ice for 10 min before centrifugation for 10 min at 4 °C. All further steps were performed according to the manufacturer’s instructions. For normalization, pellets were lysed with two-fold concentrated Laemmli buffer (100–300 µL), and the concentration of the protein lysates was determined. Colorimetric measurements were performed at 405 nm using the Infinite M Plex instrument run with the iControl Software (Tecan, Männedorf, Switzerland). Data analysis was performed using the Magellan 2.6. Software (Tecan). Finally, the measured GSH concentrations were normalized to the measured protein concentrations in the lysates of the corresponding sample.

### 2.9. ROS Measurement/DCFDA Staining

Reactive oxygen species levels were determined using the cell-permeable fluorogenic probe Chloromethyl-2′,7′-dichlorofluorescein diacetate (CM-H_2_DCFDA, Invitrogen, C6827, Darmstadt, Germany) according to the manufacturer’s instructions. PDAC cells grown in 12-well plates were incubated with 500 µL of CM-H_2_DCFDA staining solution (A818-6: 2 µM for 10 min, T3M4: 5 µM for 15 min) at 37 °C. Following the staining, cells were washed with prewarmed PBS, trypsinized with 500 µL Trypsin/EDTA for 5 to 10 min, and then collected. Pure fetal calf serum was added at 1:10 volume to neutralize the trypsin and to sequester residual dye. Stained cells were then directly measured by flow cytometry using the BD FACSVerse™ (Becton Dickinson, East Rutherford, NJ, USA).

### 2.10. Luciferase Assay

To study the activity of NRF2, PDAC cells were submitted to transfection using the Effectene^®^ transfection reagent kit and the CCS-5020 Cignal reporter assay kit (Qiagen, 336841). Cells were transfected with a reporter vector containing a firefly luciferase gene either under the control of an antioxidant responsive element (ARE) or under the control of a basal promoter element without additional transcriptional response elements, serving then as a negative control. Both reporter vectors contained a constitutively expressed noninducible Renilla luciferase gene. Cell lysates were prepared and measured using the Dual-Luciferase Reporter^®^ Assay System (Promega, Walldorf, Germany) and the software Magellan 2.6. (Tecan) implemented in an Infinite M Plex instrument. Luminescence intensities were calculated by normalizing the luciferase signal from both the control and the ARE-pretransfected cells to the Renilla luciferase signal. Finally, the specific luminescence signal was determined by normalizing the luminescence signal detected in the cells transfected with the ARE-containing vectors to the signal detected in the cells transfected with the control vectors.

### 2.11. Caspase-3/7 and MTS Assay

Caspase-3/7 activity was measured using a Caspase-Glo^®^ assay (Promega, Mannheim, Germany) according to the manufacturer’s instructions and as described [53]. Samples were measured in duplicates by fluorometry (Infinite M Plex, Tecan) and the resulting values were normalized to the respective protein concentration. MTS assay using the CellTiter 96^®^AQ_ueous_ (Promega) was conducted on cells grown on 24-well plates following the manufacturer’s instructions. Viable cell numbers indicated by MTS optical density at 490 nm were analyzed at different time points using an Infinite M Plex (Tecan) instrument.

### 2.12. Patients and Tissues

Snap-frozen pancreatic tissues were obtained from patients during surgery and their conservation was conducted by TriBank, UKSH Campus Kiel, and the Section for Translational Surgical Oncology & Biobanking, Department of Surgery, University of Lübeck (ethical approval IDs: D400/14 and 16-281). Only PDAC patients with a tumor disease pathologically staged T3N1M0 were included [55].

### 2.13. Immunohistochemical Staining

Consecutive cryostat sections (6 µm) from snap-frozen human PDAC tissues (n = 16) were mounted on uncovered glass slides and air-dried overnight at room temperature. For immunostaining of MCT1 and MCT4, slides were fixed in chilled acetone (Merck, Darmstadt, Germany) for 10 min and air-dried again for 10 min. Then, slides were washed in phosphate-buffered saline. To avoid nonspecific binding, sections were treated with 4% bovine serum albumin (Serva, Heidelberg, Germany) for 20 min, followed by overnight incubation at 4 °C with the MCT1 (HPA071055, Sigma) or MCT4 (HPA021451, Sigma) primary antibody at 1:100 dilution in 1% bovine serum albumin/phosphate-buffered saline. For immunostaining of glutamine, dried slides were incubated with a crosslinking solution (1:100, StainPerfect Immunostain Kit A, Immusmol, Bordeaux, France) for 5 min at room temperature followed by three washing steps in StainPerfect washing solution 1. Then, slides were further treated following the protocol for frozen sections (StainPerfect Immunostain Kit A, Immusmol) until overnight primary antibody incubation at 4 °C using a rabbit glutamine antibody (Abcam, ab9445) at 1:100 dilution in StainPerfect antibody diluent. After washing the antiglutamine-treated slides three times with StainPerfect washing solution 2, all slides were washed three times in phosphate-buffered saline and then treated with antirabbit peroxidase conjugates (HRP Boost rabbit, Cell Signaling) for 30 min at room temperature. Then, sections were washed three times in phosphate-buffered saline followed by peroxidase substrate reaction using the AEC peroxidase substrate kit (Abcam) according to the manufacturer’s instructions. Afterwards, sections were washed in water, counterstained in 50% haemalaun (Merck), and mounted with glycerol-gelatin. The same protocols were performed for the respective negative controls using rabbit control serum (Abcam, ab172730), showing no staining (Supplemental Figure S1). Staining was evaluated using the following scoring: 0 = no evidence of staining; 1 = moderate staining (proportion < 50%); 2 = moderate (proportion > 50%); 3 = strong staining (proportion < 50%); 4 = strong (proportion >50%).

### 2.14. Statistical Analysis

As indicated in the figure legends, normally distributed data were evaluated by a two-tailed Student’s *t*-test (Excel 2021 Software; Microsoft-Windows 11) assuming equal variance (*p* < 0.05 is considered statistically significant) and nonparametric data were evaluated by the Wilcoxon–Mann–Whitney test. All data were included in statistical analysis with no randomization or blinding. No data points were excluded.

## 3. Results

### 3.1. Upregulation of MCT1 Expression and Lactate Import by Glutamine Depletion in PDAC Cell Lines

**Figure 1:**
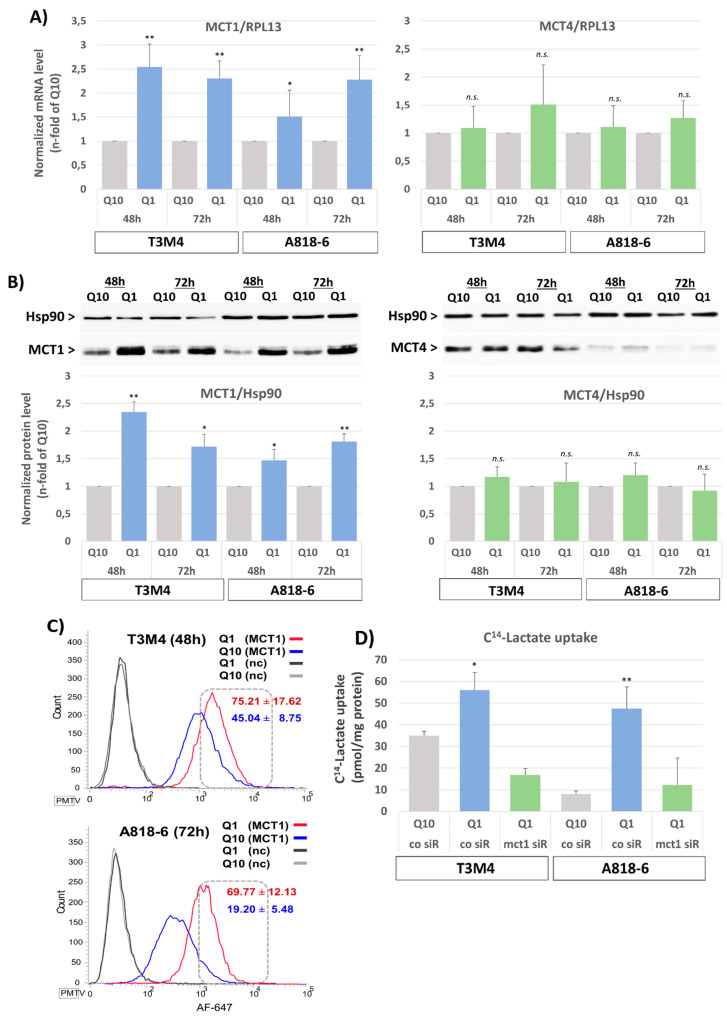
Glutamine shortage upregulates MCT1 expression. (**A**,**B**) T3M4 cells and A818-6 cells were cultured under normal glutamine supply conditions (Q10) or under glutamine shortage (Q1) for 48 h or 72 h. (**A**) mRNA levels of both MCT1 and MCT4 were measured by qPCR and normalized to the mRNA level of the housekeeper gene RPL13. Data are expressed as n-fold of Q10 and show the mean values ± SD from at least three independent experiments; * *p* < 0.05, ** *p* < 0.001, n.s., not significant. (**B**) Total cell lysates were analyzed by Western blotting to detect the expression of MCT1 and MCT4. Heat shock protein (HSP90) served as loading control. Data are expressed as the n-fold from the densitometric analysis of the western blot bands of both MCT1 and MCT4 normalized to HSP90. Mean values ± SD from at least three independent experiments are shown. * *p* < 0.05, ** *p* <0.001, n.s., not significant. Western blot figures from a representative experiment for each cell line are shown. (**C**) Cells were immunostained with an AF647-conjugated anti-MCT1 (MCT1) or control antibody (nc) and then analyzed by fluorescence flow cytometry. Histograms show the distribution of AF647 positive cells. A representative out of three independent experiments and the mean values ± SD are shown. (**D**) T3M4 and A818-6 cells were first subjected to either control (co siR) or MCT1 siRNA (MCT1 siR) treatment for 24 h (see Supplemental Figure S2) and then switched to Q10 or Q1 culture for 48 h. Cells were then submitted to the ^14^C-lactate uptake assay. Data show the specific incorporation of ^14^C-lactate normalized to the amount of protein. Mean values ± SD from three independent experiments are shown. * *p* < 0.05, ** *p* < 0.001.

The pancreatic cancer cell lines A818-6 and T3M4, expressing MCT1 at low and moderate level, respectively, and capable of adopting a reverse Warburg metabotype [39], were exposed to a normal (RPMI with 2 mM Gln) or glutamine-reduced (RPMI with 0.2 mM Gln) culture medium for various periods. In both cell lines, glutamine depletion led to an increase in MCT1 expression on both the mRNA and protein levels (Figure 1). MCT1 expression strongly increased in T3M4 cells within 48 h of starting glutamine depletion (Figure 1A,B) and then started to decline after 72 h, whereas in A818-6 cells, the effect of glutamine withdrawal on MCT1 expression was not seen earlier than 48–72 h (Figure 1A,B). By contrast, expression of the lactate exporter MCT4 remained largely unaffected in both cell lines. To verify that MCT1 surface expression is also upregulated by glutamine depletion, unpermeabilized A818-6 and T3M4 cells were analyzed by MCT1 immunostaining and subsequent fluorescence flow cytometry. As shown in Figure 1C, the fraction of T3M4 cells with high surface expression increased from 45 ± 8.8% to 75 ± 17.6% after Q1-treatment for 48 h and that of A818-6 cells increased from 19 ± 5.5% to 69.8 ± 12.1% after Q1-treatment for 72 h.

Next, it was investigated whether an increase in MCT1 expression alters lactate uptake into these two cell lines. As shown by the ^14^C-lactate uptake assay (Figure 1D), T3M4 and A818-6 cells exhibited a significantly increased uptake of ^14^C-lactate (54 versus 35 pmol/mg protein and 46 versus 6 pmol/mg protein, respectively) when glutamine was depleted for 48 h. The siRNA-mediated knock-down of MCT1 strongly reduced the effect of glutamine depletion on ^14^C-lactate import into both PDAC cell lines (Figure 1D).

### 3.2. Glutamine-Scarce Regions in Human PDAC Tissue Exhibit Greater MCT1 Expression

**Figure 2:**
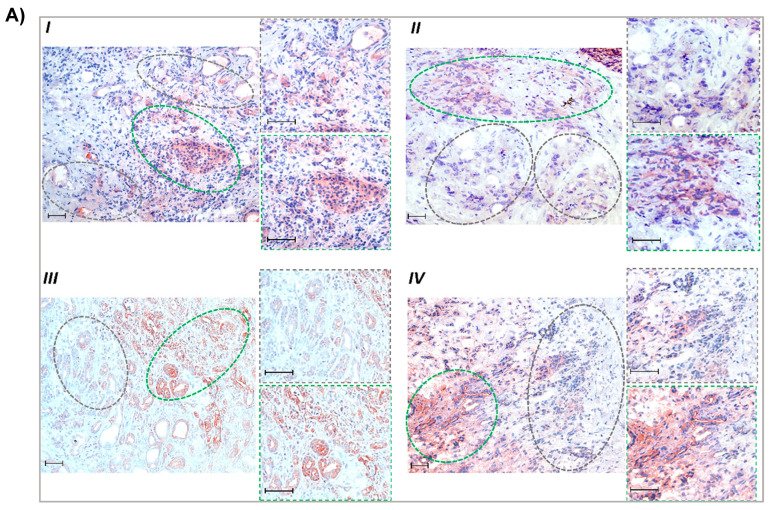

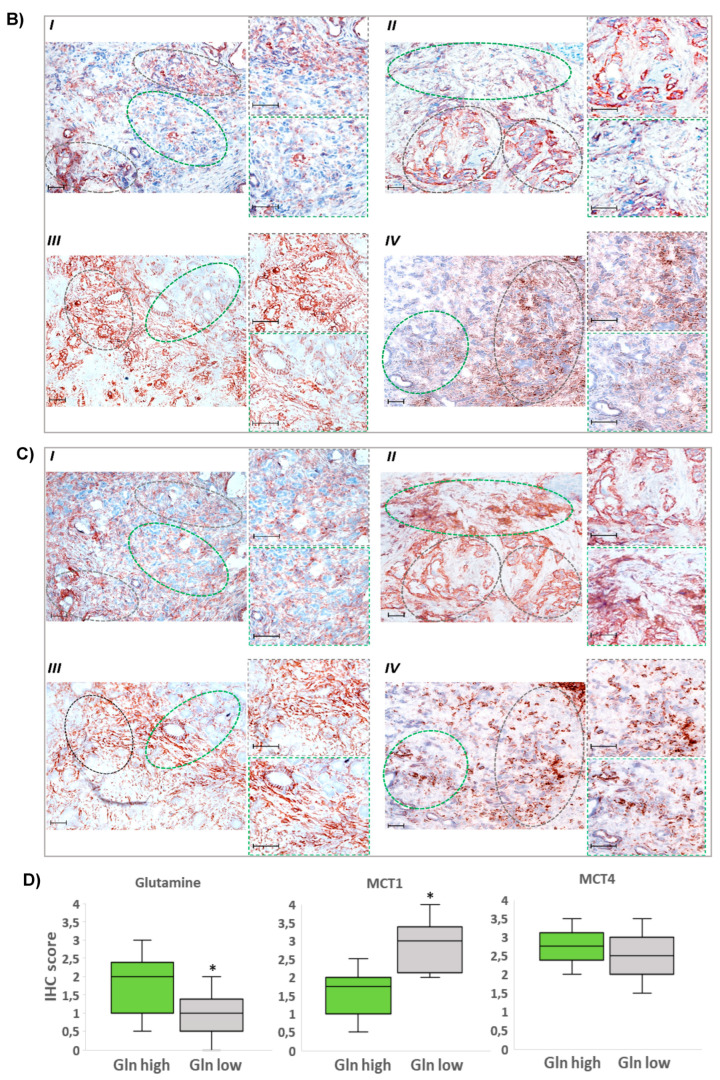
Greater MCT1 expression in glutamine-scarce areas within human PDAC tissue. Consecutive cryostat sections (6 µm) of snap-frozen specimens from PDAC patients (all classified T3N1M0) were submitted to immunohistochemistry (IHC). (**A**) Following an adapted crosslinking fixation protocol (see Section 2.13), PDAC sections were probed with a glutamine antibody. (**B**,**C**) Acetone-fixed tissue slides were probed with (**B**) MCT1 and (**C**) MCT4 antibodies. Tissue areas with low glutamine reactivity are encircled by grey-dashed lines and those with high glutamine reactivity are encircled by green-dashed lines. Staining from 4 different cases (I–IV) are shown (scale bar: 50 µm) including images at higher magnification from high- and low-glutamine-stained areas. (**D**) From immunohistochemically stained specimens (n = 16), representative microscopic fields (in total n = 31) were evaluated using IHC scoring (see Section 2.13). Boxes cover the 0.25 and 0.75 quantiles and whisker lengths cover the medial deviation; * *p* < 0.05.

Next, we investigated whether elevated MCT1 expression is associated with a lower glutamine supply in human PDAC tissues. For this purpose, immunohistochemical analysis was performed with consecutive sections of snap-frozen tumor tissue specimens from PDAC patients (Figure 2). Performing an adapted crosslinking fixation protocol, extended areas with higher glutamine immunoreactivity (Figure 2A) were detected (median IHC score = 2). These tissue regions largely contained tumor as well as stromal cells with weak or moderate expression (median score = 1.75) of MCT1 (Figure 2B).

In areas exhibiting only weak staining of glutamine (median score = 1), the tumoral expression of MCT1 was much greater (median score = 3), whereas MCT4 expression (Figure 2C) was not different between glutamine-positive and -negative regions (median score = 2.75 and 2.5, respectively) on both tumor and stromal cells. IHC scores are shown in Figure 2D. Thus, glutamine-scarce tumor regions are characterized by a more reverse Warburg-like metabotype exhibiting stronger MCT1 expression, while in glutamine-rich regions, MCT4 expression prevails.

### 3.3. The Inducing Effect of Glutamine Depletion on MCT1 Expression in T3M4 and A818-6 Cells Depends on Oxidative Stress and Nrf2 Activation

Recently, it was shown that MCT1 is a target gene of the antioxidant transcription factor Nrf2 [56], a key regulator of the cellular antioxidant response [57]. This prompted us to investigate the possible relation between Nrf2 activation under the loss of glutamine-dependent redox homeostasis and the induced MCT1-driven lactate import.

**Figure 3:**
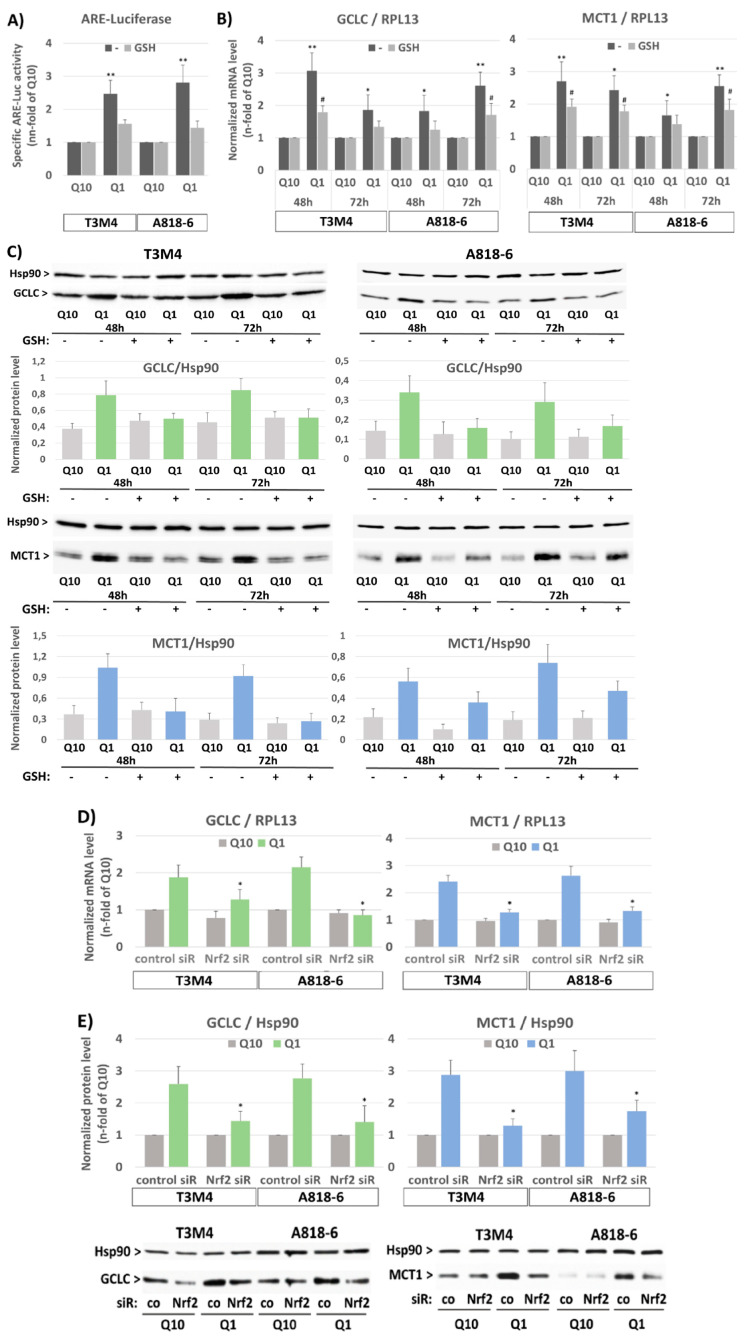
The induction of MCT1 expression on the protein level under glutamine shortage depends on oxidative-stress-induced NRF2 activation in PDAC cells. (**A**) T3M4 and A818-6 cells were transfected with either luciferase control or ARE vectors for 24 h followed by culture under normal glutamine (Q10) or low glutamine (Q1) supply for 24 h, either in the absence (-) or presence of 1.5 mM glutathione (GSH). Then, cell lysates were submitted to luminescence measurement. Data are expressed as the n-fold of the adjusted firefly/Renilla signal under ARE and normalized to control (mean values ± SD from six independent experiments are shown; ** *p* < 0.001). (**B**,**C**) T3M4 and A818-6 cells were cultured under normal glutamine (Q10) or low glutamine (Q1) supply for 48 h and 72 h, either in the absence (-) or presence of 1.5 mM glutathione (GSH). (**B**) mRNA levels of GCLC and MCT1 were analyzed by qPCR using RPL13 as housekeeper mRNA for normalization. Data represent the n-fold of Q10 and mean values ± SD from four independent experiments; * *p* < 0.05 and ** *p* <0.001 compared to Q10; ^#^ *p* < 0.05 compared to without (-). (**C**) Protein levels of GCLC and MCT1 were analyzed by Western blot using HSP90 as a loading control. After densitometric analysis of band intensities, GCLC and MCT1 were normalized to HSP90. Mean values ± SD from three independent experiments are shown. Western blot figures from a representative experiment for each cell line are shown. (**D**,**E**) T3M4 and A818-6 cells were treated first with either control or NRF2 siRNA (siR) (see Supplemental Figure S3) followed by 48 h culture under normal (Q10) or low (Q1) glutamine supply. (**D**) mRNA levels of GCLC and MCT1 were analyzed by qPCR using RPL13 as housekeeper mRNA for normalization. Mean values ± SD from four independent experiments are shown; * *p* < 0.05 compared to control siRNA. (**E**) Protein levels of GCLC and MCT1 were analyzed by Western blot using HSP90 as a loading control. After densitometric analysis of the band intensities, GCLC and MCT1 were normalized to HSP90. Mean values ± SD from four independent experiments are shown; * *p* < 0.05 compared to control siR. Western blot figures depicted below show one representative experiment on each cell line.

For the analysis of Nrf2 activation, ARE luciferase assays were conducted on T3M4 and A818-6 cells. A significant increase in Nrf2-induced reporter gene activity was detected in both cell lines within 24 h of glutamine depletion (Figure 3A). To verify that Nrf2 activation depends on the loss of redox maintenance due to the absence of glutamine, the cellular antioxidant GSH was added to the glutamine-starved cells. Under this condition, the inducing effect of glutamine withdrawal was abrogated (Figure 3A). An increased expression of the established Nrf2 target gene GCLC within 48–72 h of glutamine withdrawal confirmed the Nrf2-dependent antioxidant response in both cell lines (Figure 3B,C). Again, in the presence of GSH, the inducing effect of glutamine withdrawal on GCLC expression was much less pronounced. Intriguingly, the induced MCT1 expression along with glutamine depletion was similarly affected by GSH in T3M4 and A818-6 cells (Figure 3B,C). Next, MCT1 expression during glutamine depletion was analyzed in T3M4 and A818-6 cells subject to siRNA-mediated Nrf2 knock-down. As shown in Figure 3D,E, the increased MCT1 expression following glutamine withdrawal was diminished by the Nrf2 knock-down in both cell lines. Similarly, the induction of GCLC expression was reduced (Figure 3D,E). In contrast to MCT1, the expression of MCT4 was not affected by the Nrf2 knock-down or addition of GSH.

### 3.4. Lactate Protects T3M4 and A818-6 Cells from Glutamine-Depletion-Induced ROS Stress Depending on MCT1 Expression

**Figure 4:**
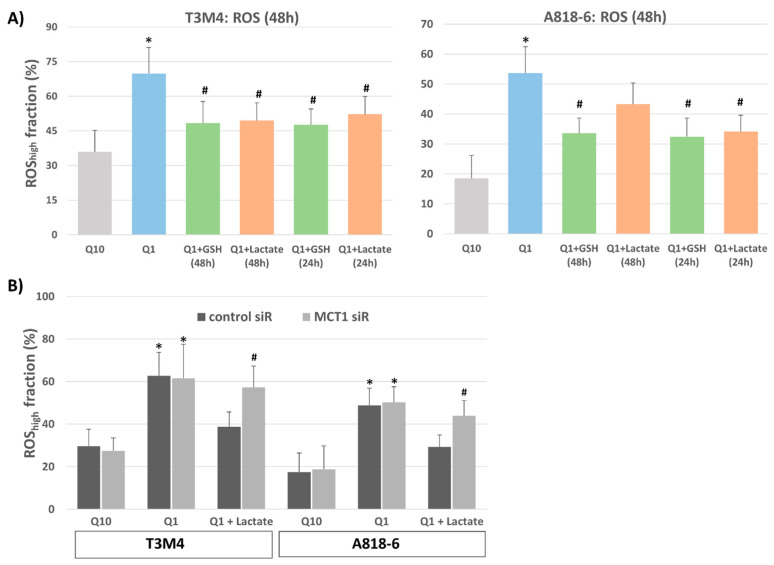
Lactate protects T3M4 and A818-6 cells from glutamine-depletion-induced ROS stress depending on MCT1 expression. (**A**) T3M4 and A818-6 cells were cultured under normal glutamine (Q10) or low glutamine (Q1) supply for 48 h, either in the absence or presence of 1.5 mM glutathione (GSH) or 20 mM lactate given during the same period (48 h) or 24 h later. Then, cells were DCFDA-stained and analyzed by flow cytometry (see Supplemental Figure S4). Those fluorescence signals >20-fold greater than the background signal (ROS_high_) were quantified as the percentage of stained cells. Mean values ± SD from six independent experiments are shown; * *p* < 0.05 compared to Q10; ^#^ *p* < 0.05 compared to Q1 without GSH or lactate. (**B**) T3M4 and A818-6 cells were treated first with either control or MCT1 siRNA (siR) (see Supplemental Figure S2) followed by 48 h culture under normal glutamine (Q10) or low glutamine (Q1) supply, either in the absence (-) or presence of 20 mM lactate given during the same period or 24 h later. Then, analysis of DCFDA-stained cells was performed as described above. Mean values ± SD from four independent experiments are shown; * *p* < 0.05 compared to Q10; ^#^ *p* < 0.05 compared to control siR.

Given the essential role of glutamine in the maintenance of cellular redox balance, we wondered whether lactate could compensate for the loss of glutamine and its accompanying increase in the intracellular ROS level. When analyzing T3M4 or A818-6 cells stained with the ROS-detecting dye CM-H2-DCFDA and quantified by flow cytometry, it could be seen that glutamine-starved cells exhibited stronger staining (Figure 4A). In T3M4 and A818-6 cells, the fraction of cells exhibiting high DCFDA staining increased from 36% to 70% and 18% to 53%, respectively, when cultured under glutamine depletion for 48 h.

The addition of 20 µM lactate to T3M4 cells together with (48 h) or 24 h after glutamine withdrawal reduced ROS production, as indicated by the drop in the highly DCFDA-stained fraction from 70% to 49% and 52%, respectively (Figure 4A). These effects of lactate were similar to those seen with glutathione (GSH) that were analyzed for comparison (Figure 4A). The addition of GSH reversed the increase in ROS level upon glutamine withdrawal at both time points (drop in highly DCFDA-stained fraction to 49% and 48%, respectively).

In contrast to T3M4 cells, the addition of lactate to A818-6 cells affected ROS staining during glutamine depletion only when taking place afterwards (24 h), as shown by the drop in the highly DCFDA-stained fraction from 53% to 34%. Its simultaneous addition (48 h) only had a moderate effect on cellular ROS level, as the amount of highly DCFDA-stained A818-6 cells only changed from 53% to 44% (Figure 4A). The addition of GSH, for comparison, reduced the amount of highly DCFDA-stained A818-6 cells from 53% to 34% and 32%, respectively, when added together with (48 h) and 24 h after glutamine withdrawal. These time-dependent differences in lactate effects in the two cell lines could be explained by the fact that A818-6 cells only exhibited low expression of MCT1 compared with T3M4 cells. Instead, higher MCT1 expression was only seen following glutamine withdrawal (see above).

The effect of lactate addition on elevated ROS levels in glutamine-starved T3M4 cells and A818-6 cells was abolished by the siRNA-mediated knock-down of MCT1 (Figure 4B). Thus, in T3M4 cells, the reducing effect of lactate added simultaneously with the glutamine withdrawal for 48 h was not seen upon MCT1 siRNA pretreatment (61% and 59%, respectively, of the highly DCFDA-stained cell fraction) when compared with control-siRNA pretreated cells (63% and 39%, respectively, of highly DCFDA-stained cell fraction). Likewise, lactate addition 24 h after glutamine withdrawal was almost ineffective in A818-6 cells subjected to MCT1 siRNA pretreatment (51% and 47%, respectively, of highly DCFDA-stained cell fraction) when compared with control siRNA-pretreated cells (49% and 29%, respectively, of the highly DCFDA-stained cell fraction).

### 3.5. MCT1-Driven Lactate Uptake Reconstitutes GSH Content in Glutamine-Starved PDAC Cells

**Figure 5:**
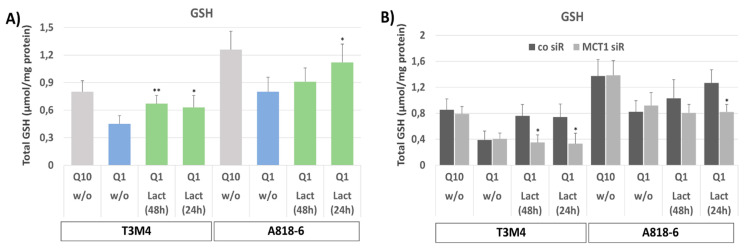
Lactate treatment compensates for the loss of GSH synthesis in glutamine-starved PDAC cells. Both (**A**) T3M4 and A818-6 cells were cultured with Q10 or Q1, the latter either without (w/o) lactate or with lactate given either in parallel with Q1 culture (48 h) or 24 later. Afterwards, cells were lysed and submitted to colorimetric glutathione assay. Data represent the mean values ± SD of total GSH level from three independent experiments. ** *p* < 0.001 & * *p* < 0.05 compared with Q1 without lactate. (**B**) Both cell lines were also submitted to MCT1 knock-down (MCT1 siR) or not (co siR) (see Supplemental Figure S2) before further culture and sample collection as described above. Afterwards, cells were lysed and submitted to colorimetric glutathione assay. Data represent the mean values ± SD of total GSH level from three independent experiments; * *p* < 0.05 compared with control siR.

By analyzing the total cellular GSH levels (Figure 5A), it can be seen that glutamine-starved (for 48 h) T3M4 and A818-6 cells contained lower amounts of GSH compared to cells cultured under normal conditions (0.43 versus 0.78 µmol/mg protein and 0.79 versus 1.23 µmol/mg protein, respectively).

When lactate was added either directly after glutamine withdrawal (48 h) or 24 h later, the decrease in GSH level by glutamine starvation was less pronounced in T3M4 cells at both time points (0.61 and 0.58 µmol/mg protein, respectively). In A818-6 cells, only the later addition of lactate affected the GSH level (1.12 µmol/mg protein) but not its earlier addition (0.83 µmol/mg protein GSH). Again, these time-dependent differences in the effects of lactate addition can be explained by the distinct basal expression levels of MCT1 in T3M4 and A818-6 cells. In the latter cells, MCT1 expression needs to be induced by glutamine starvation first. By contrast, the amount of oxidized glutathione (GSSG), making up only 1–2% of total GSH, was not affected by the different treatments. The knock-down of MCT1 in T3M4 and A818-6 cells abolished the increasing effect of lactate addition on the declined GSH level in both cell lines when subjected to glutamine starvation (Figure 5B).

### 3.6. Lactate Protects T3M4 and A818-6 Cells from Glutamine Depletion-Induced Cell Death Depending on MCT1 Expression

**Figure 6:**
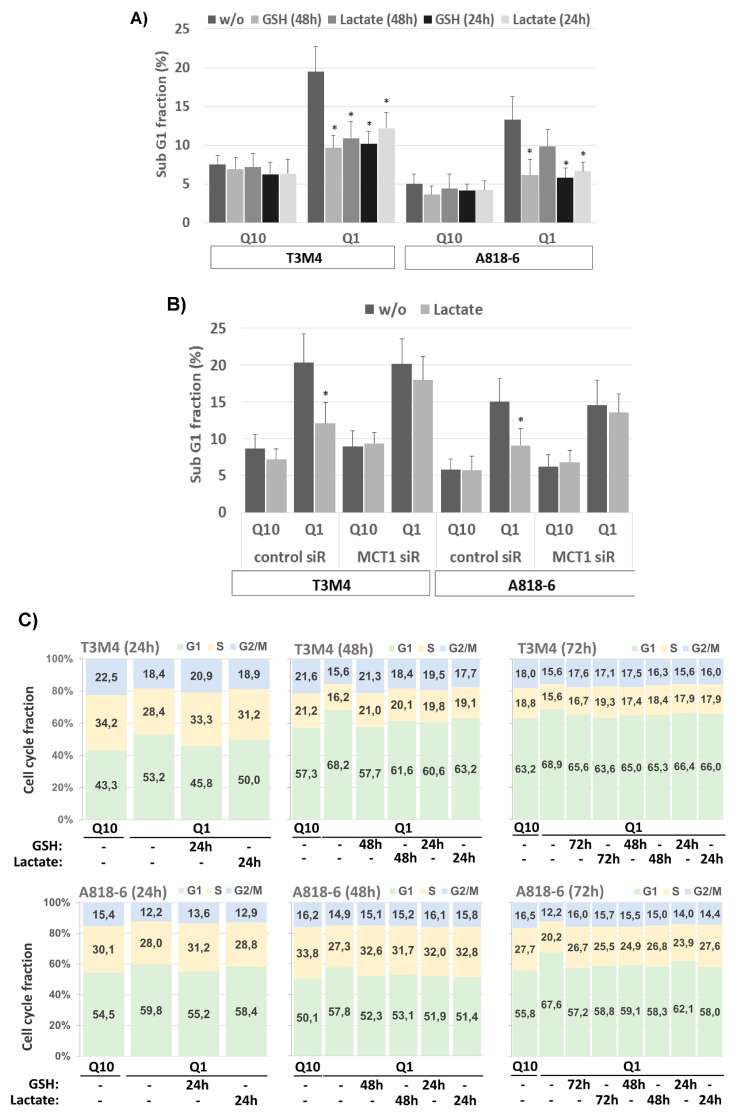

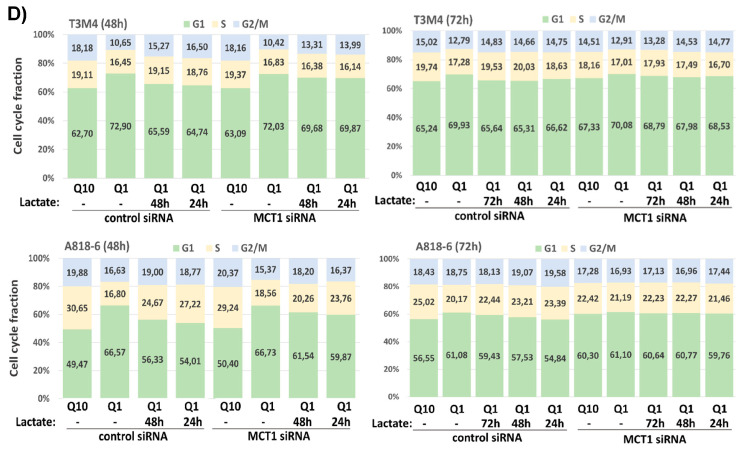
Lactate protects PDAC cells from cell death and cell cycle arrest under glutamine shortage depending on MCT1. (**A**) T3M4 and A818-6 cells cultured with Q10 or Q1 for 48 h in the absence or presence of 1.5 mM GSH or 20 mM lactate added in parallel (48 h) or 24 h later were collected and stained with PI and analyzed by flow cytometry (see Supplemental Figure S5). The subG1 fraction containing apoptotic cells was quantified. Mean values from six independent experiments are shown; * *p* < 0.05 compared to “w/o”. (**B**) T3M4 and A818-6 cells transfected with control or MCT1 siRNA (see Supplemental Figure S2) were cultured with Q10 or Q1 for 48 h either alone or with lactate addition in parallel (T3M4) or 24 h later (A818-6). PI-stained cells were quantified for the subG1 fraction. Mean values from four independent experiments are shown; * *p* < 0.05 compared to control siRNA. (**C**) T3M4 and A818-6 cells cultured with Q10 or Q1 for 24 h, 48 h, or 72 h in the absence or presence of 1.5 mM GSH or 20 mM lactate added in parallel or later, as indicated, were collected and stained with PI and analyzed by flow cytometry (see Supplemental Figure S5). The G1, S and, G2/M cell cycle fractions were quantified. Mean values from six independent experiments are shown. (**D**) T3M4 and A818-6 cells pretreated with control or MCT1 siRNA (see Supplemental Figure S2) were cultured with Q10 or Q1 for 48 h or 72 h in the absence or presence of 20 mM lactate added in parallel or later, as indicated. PI-stained cells were quantified for the G1-, S-, and G2/M-cell cycle fractions. Mean values from four independent experiments are shown.

Next, it was investigated whether glutamine depletion leads to cell death of T3M4 and A818-6 cells. As shown by PI staining and flow cytometry, the subG1 fraction of T3M4 and A818-6 cells increased from 7.5% to 19.5% and 5.1% to 13.3%, respectively, after a 48 h glutamine withdrawal (Figure 6A). The addition of GSH or lactate reduced the induction of cell death during glutamine depletion, as shown by the drop in subG1 fractions (Figure 6A).

In T3M4 cells, the subG1 fraction decreased to 10.9% and 12.2% during glutamine depletion when lactate was added simultaneously (48 h) and afterwards (24 h), respectively. In A818-6 cells, treatment with lactate reduced the subG1 fraction to a lesser extent when added simultaneously with glutamine withdrawal (8.9%), whereas the addition of lactate 24 h afterwards significantly decreased the subG1 fraction to 6.0% (Figure 6A). Again, these time-dependent differences in the effects of lactate addition in T3M4 and A818-6 cells, respectively, reflect the differences in basal MCT1 expression (see above).

To confirm the involvement of MCT1, T3M4 and A818-6 cells were treated with control siRNA or MCT1 siRNA followed by a glutamine-scarce culture for 48 h either with or without lactate addition. As shown in Figure 6B, the effect of lactate addition during the 48 h glutamine withdrawal and 24 h afterwards on the fraction of subG1-phase cells of MCT1 siRNA-pretreated T3M4 and A818-6 cells, respectively, was abrogated as compared to cells pretreated with control siRNA.

### 3.7. Lactate Protects T3M4 and A818-6 Cells from Glutamine Depletion-Induced Cell Cycle Arrest Depending on MCT1 Expression

Cell cycle analysis after PI staining and flow cytometry further revealed that the G1 fraction of both T3M4 and A818-6 cells increased during glutamine starvation while the number of cells in the S- and G2/M-phases was reduced (Figure 6C; see also Supplemental Table S1). Thus, 53.22%, 68.15%, and 68.87% of T3M4 cells were in the G1-phase after glutamine withdrawal for 24 h, 48 h, and 72 h, respectively, compared to 43.26%, 57.28%, and 63.19%, respectively, of T3M4 cells in G1-phase after culture with normal medium. Similarly, a higher fraction of A818-6 cells was arrested in the G1-phase over 24 h, 48 h, and 72 h after treatment under glutamine starvation (59.83%, 57.72%, and 67.58%, respectively) compared to the culture in normal medium (54.51%, 50.07%, and 55.80%, respectively). Like GSH, when added along with or after glutamine depletion, lactate treatment partially released T3M4 and A818-6 cells from the G1-phase in a time-dependent manner (Figure 6C). In T3M4 cells, lactate addition most effectively affected the G1-, S-, or G2/M-phases if added over the entire period of glutamine withdrawal (24 h, 48 h, and 72 h, respectively). By contrast, during glutamine starvation for 48 h and 72 h, lactate addition at later time points (24 h and 24 h or 48 h after glutamine withdrawal, respectively) was less effective. In A818-6 cells, lactate was able to resume the cell cycle only under glutamine starvation for 48 h and 72 h. This resuming effect on G1-, S-, or G2/M-phases was most pronounced in A818-6 cells during glutamine starvation for 72 h when lactate was added 24 h after glutamine withdrawal. To confirm the involvement of MCT1, T3M4 and A818-6 cells were treated with control siRNA or MCT1 siRNA followed by a glutamine-scarce culture for 48 h and 72 h either with or without lactate addition at different time points. As shown in Figure 6D and Supplemental Table S2, the effect of lactate addition on the G1-, S-, and G2/M-phases of MCT1 siRNA-pretreated T3M4 or A818-6 cells was abrogated as compared to cells pretreated with control siRNA.

### 3.8. MCT1-Driven Lactate Uptake Favors Resistance of PDAC Cells against ASCT2 and GLS Inhibitors

**Figure 7:**
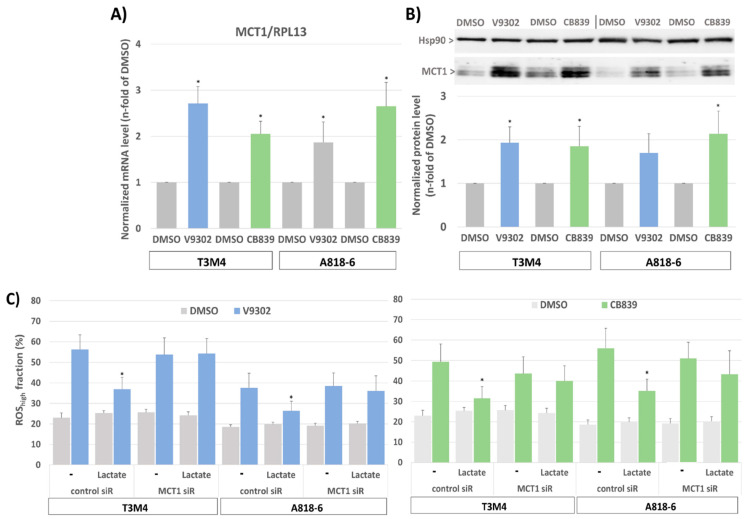
MCT1 expression in PDAC cells increases after glutamine uptake and glutaminase inhibition and provides protection from ROS stress by lactate. (**A**) T3M4 and A818-6 cells treated with the ASCT2 inhibitor V9302 (2 µM) or the glutaminase-1 inhibitor CB839 (10 µM) or the vehicle (DMSO) were analyzed by (**A**) qPCR of MCT1 using RPL13 as housekeeper mRNA for normalization. Mean values from four independent experiments are shown; * *p* < 0.05 compared to DMSO, or (**B**) Western blot of MCT1 using HSP90 as a loading control for normalization. Mean values from four independent experiments are shown; * *p* < 0.05 compared to DMSO. (**C**) T3M4 and A818-6 cells pretreated with control or MCT1 siRNA were treated with V9302, CB839, or DMSO for 48 h either in the absence (w/o) or presence of 20 mM lactate for 48 h and 24 h, respectively. Then, cells were DCFDA-stained and analyzed by flow cytometry (see Supplemental Figure S6). Those fluorescence signals >10-fold greater than the background signal (ROS_high_) were quantified as the percentage of stained cells. Mean values ± SD from six independent experiments are shown; * *p* < 0.05 compared to (-).

Since targeting glutamine metabolism has been recently recognized as a novel and powerful strategy in cancer therapy [58], including PDAC, several drugs have been developed and introduced in clinical trials targeting glutamine transporters such as the ASCT2(SLC1A5) inhibitor V9302 or the glutaminase-1 (GLS1) inhibitor CB839 [59,60]. Like glutamine depletion, the treatment of T3M4 and A818-6 cells with both drugs for 48 h led to an elevated expression level of MCT1 (Figure 7A,B) as well as an increase in cellular ROS levels (Figure 7C). Whilst V9302 was more effective in T3M4 cells, CB839 was more effective in A818-6 cells. Thus, V9302 and CB839 increased the ROS level in T3M4 cells from 23% to 56% and 49%, respectively, and in A818-6 cells from 19% to 38% and 56%, respectively. In both cell lines, treatment with lactate reduced drug-induced ROS accumulation, an effect that was abrogated by the knock-down of MCT1 (Figure 7C).

**Figure 8:**
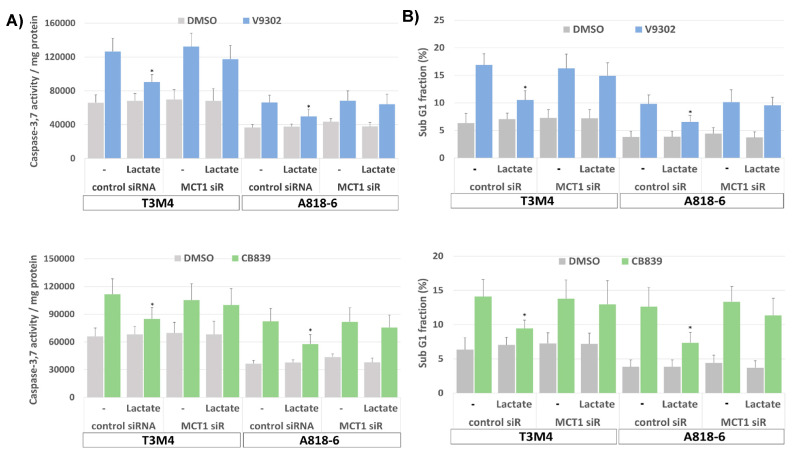

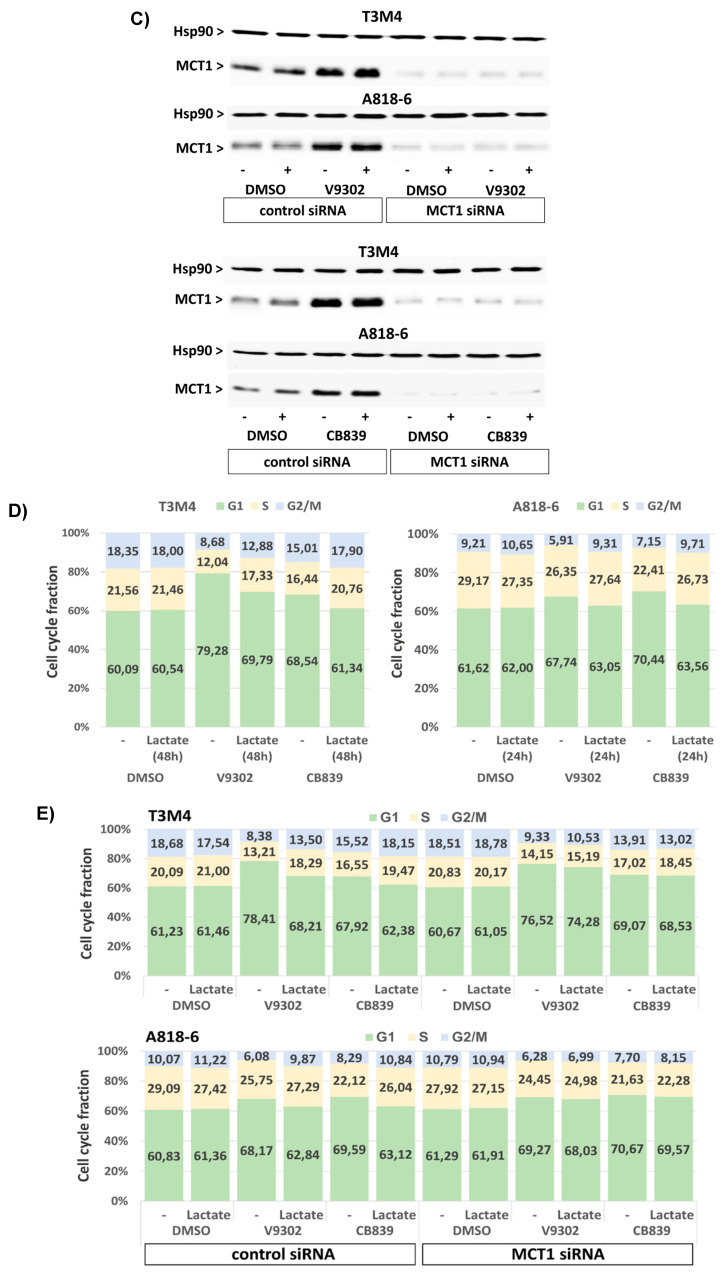

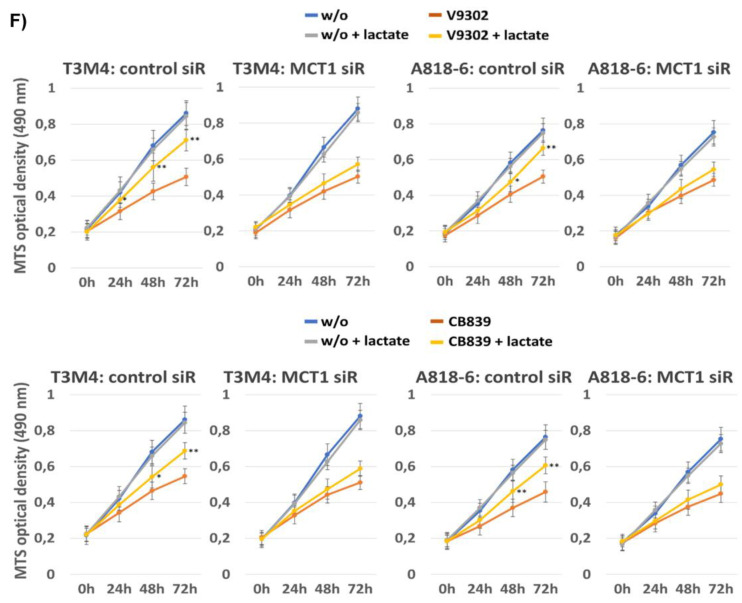
Lactate protects PDAC cells from V9302- or CB839-induced cell death and cell cycle arrest depending on MCT1. T3M4 and A818-6 cells subject either to (**A**–**C**,**E**,**F**), control or MCT1 siRNA transfection for 24 h or (**D**) no siRNA pretreatment were treated with V9302 (2 µM), CB839 (10 µM), or DMSO for 48 h (**A**–**E**) or 72 h (**F**) either in the absence (-) or presence of 20 mM lactate. Then, cells were either collected (**A**–**E**) and (**A**) subjected to Caspase-3,7 assay or (**B**,**D**,**E**) stained with PI and analyzed by flow cytometry (see Supplemental Figure S7), or (**C**) cell lysates were obtained and subjected to MCT1 Western blot using HSP90 as loading control, or (**F**) cells were analyzed by MTS assay at the indicated periods. (**A**) Caspase-3,7 activity was normalized to protein content and (**B**) subG1 fractions containing apoptotic cells were quantified. Mean values from four independent experiments are shown; * *p* < 0.05 compared to “-”. (**D**,**E**) PI-stained cells were quantified for the G1-, S-, and G2/M-cell cycle-fractions. Mean values from four independent experiments are shown. (**F**) MTS optical density was analyzed; data represent the mean of six independent experiments; ** *p* < 0.02 & * *p* < 0.05 compared to V9302 or CB839 without lactate.

Moreover, treatment with V9302 and CB839 induced the cell death of T3M4 and A818-6, as shown by the enhanced caspase-3,7 activity and the increased subG1 fraction of PI-stained cells (Figure 8A,B). Thus, V9302 and CB839 treatment of T3M4 cells for 48 h elevated caspase-3,7 activity 2-fold and increased the subG1 fraction from 6.4% to 17.1% and 14.3%, respectively. V9302 and CB839 treatment of A818-6 cells for 48 h resulted in 2- and 3-fold elevated caspase-3,7 activity, respectively, while the subG1 fraction increased from 3.4% to 9.9% and 13.2%, respectively. This cell-death-inducing effect by the two inhibitors was diminished in T3M4 cells (elevation of caspase-3,7 activity less than 1.3-fold and increase in subG1 to 10.7% and 8.8%, respectively) and A818-6 cells (elevation of caspase-3,7 activity less than 1.2-fold and increase in subG1 to 6.3% and 7.4%, respectively) by the addition of lactate together with (48 h) and after (24 h) the administration of the drugs, respectively. The siRNA-mediated knock-down of MCT1 (verified by Western blot, Figure 8C) abrogated the effect of lactate on V9302- and CB839-induced cell death (Figure 8A,B), as shown by the still-elevated caspase-3,7 activity and increased subG1 fractions in the drug-treated cells. As shown in Figure 8D and Supplemental Table S3, V9302 and CB839 led to a cell cycle arrest in both cell lines. After 48 h of treatment, the G1-phase fraction of T3M4 cells increased from 60.1% to 79.3% and 68.5%, respectively, and that of A818-6 cells increased from 61.6% to 67.7% and 70.4%, respectively. The addition of lactate to T3M4 cells for the entire period of drug administration and to A818-6 cells 24 h after it reduced the increasing effect of V9302 (69.8% and 63.1%, respectively) and CB839 (61.3% and 63.6%, respectively).

Again, the knock-down of MCT1 abrogated the effect of lactate on V-9302- and CB839-induced cell cycle arrest (Figure 8E and Supplemental Table S4), as shown by the still-elevated number of drug-treated cells in the G1-phase. Moreover, upon treatment with V9302 and CB839, the number of living T3M4 and A818-6 cells was strongly reduced over a period of 24–72 h, as shown by MTS assay (Figure 8F). In the presence of lactate, the growth-suppressing effect of both drugs was already diminished in T3M4 cells after 24 h and in A818-6 cells after 48 h. The knock-down of MCT1 abolished the inhibitory effect of lactate on drug-induced growth suppression in both cell lines (Figure 8F). Overall, the induction of MCT1-dependent lactate uptake enables PDAC cells to overcome growth suppression caused by inhibiting glutamine uptake/metabolism.

## 4. Discussion

Cancer cells are particularly addicted to the amino acid glutamine because it essentially feeds a variety of metabolic pathways underlying proper growth and survival [10,11,12]. In fact, glutamine is described as the most depleted amino acid in PDAC, emphasizing its high demand in PDAC cells [61,62,63]. Thus, regional glutamine deficiency is detectable within PDAC tissues when compared with adjacent benign tissue [61,64]. Representing an essential nutrient and the most rapidly consumed one by cancer cells besides glucose, glutamine metabolism has been also found to be interconnected with that of glucose. Based on accumulating evidence for the mutual dependence of glycolysis and glutamine metabolism [13,65], one can speculate that glutamine deficiency in PDAC could result in a metabolic pressure initiating the reverse Warburg metabolism as a compensatory effect. Previous studies showed that glucose is required for glutamine uptake and that glucose withdrawal could lead to a tenfold reduction in glutamine metabolism, as observed in hematopoietic cells and B cells [50,51,52,53,54,55,56,57,58,59,60,61,62,63,64,65,66,67]; one can speculate that glutamine deficiency in PDAC could result in metabolic pressure initiating the reverse Warburg metabolism as a compensatory effect.

In the present study, we showed that glutamine-starved PDAC cells highly upregulate their lactate uptake by increasing the expression of MCT1, accompanied by a restoration of the cellular redox homeostasis that is otherwise disrupted during glutamine starvation [17,18]. The underlying sensitivity of PDAC cells to low glutamine supply and the urgency to reprogram their metabolism to retrieve compensatory nutrients such as lactate can be explained by the high reliance of PDAC cells on glutamine as a major carbon and nitrogen source to fulfill metabolic and biosynthetic requirements [67,68] as well as to feed the GSH-mediated redox system. In accordance with these essential roles of glutamine in cancer cell metabolism, the inhibition of its uptake or enzymatic conversion by glutaminases effectively impacts PDAC cell proliferation. Given the important role of glutamine in GSH-dependent redox homeostasis [17,18,69], we further unveiled the mechanism of action behind the increased MCT1-mediated lactate uptake in PDAC cells under glutamine starvation. Accordingly, we were able to demonstrate that the increased MCT1 expression in glutamine-starved PDAC cells depends on the activation of Nrf2. This finding goes in line with the established role of Nrf2 in the protection of cells from oxidative stress. Thus, it was shown not only that Nrf2 essentially controls the antioxidant glutathione (GSH) pathway [70] but also that Nrf2 activation by oxidative stress leads to an upregulation of MCT1-driven lactate uptake in cancer cells [56].

In fact, the Nrf2-dependent upregulation of MCT1 expression allows PDAC cells to compensate for the drop in anaplerosis via glutamate when glutamine availability is limited. Thus, the balance of glutamine utilization towards glutamate either entering the citric acid cycle (CAC) via alpha-ketoglutarate or feeding GSH synthesis (by GCLC, etc.) is critically compromised under glutamine starvation [71]. If no compensatory nutrient is available, glutamate anaplerosis competes with GSH synthesis, leading to a delicate drop in GSH-dependent redox homeostasis, which limits the growth and survival of cancer cells [47,71,72]. Through the forced uptake of other anaplerotic metabolites—such as lactate due to elevated MCT1 expression—and their introduction into the TCA, the cells are released from the need to incorporate glutamate into the TCA [47,73]. Consequently, the limited availability of glutamine no longer stresses the balance of glutamate anaplerosis versus its usage for GSH synthesis. Under this condition, glutamate derived from glutamine (via GLS) could serve as a substrate for generating sufficient amounts of GSH to a much greater extent, thereby restoring the redox homeostasis that was lost upon glutamine starvation [71,72,73]. In this fashion, PDAC cells are able to overcome not only oxidative stress, as indicated by the drop in ROS levels, but also growth suppression due to glutamine starvation, as indicated by the re-entry of the cell cycle and decreased apoptosis.

Another mechanism of the antioxidant- and tumor-promoting effect of MCT1 has recently been reported in melanoma cells [35]. Here, MCT1-driven lactate import is suggested to cause a lowering of intracellular pH, which inhibits the glycolytic enzyme phospho-fructokinase-1 (PFK1). Consequently, the accumulated glucose-6P is redirected to the pentose phosphate pathway, generating more NADPH that is used for GSH reduction and provides greater oxidative stress resistance [35,74]. In our experiments, however, we did not detect alterations in the reduced state of GSH, suggesting that in PDAC cells, the balance of glutamate utilization towards GSH synthesis is of major importance, as described in other tumor entities such as small-cell lung, breast, and hepatocellular carcinoma, as well as lymphoma [71,72,73,74,75,76].

MCT1-driven compensation for glutamine starvation is of great relevance in view of the efficacy of therapeutic strategies targeting glutamine metabolism in which MCT1-mediated lactate provides an efficient resistance mechanism in PDAC cells. Here, the inhibition of the glutamine transporter ASCT2 by V9302 [59] or of glutaminase activity by GLS inhibitors such as CB839 [60] forced oxidative stress in T3M4 and A818-6 PDAC cells and suppressed their growth. Notably, MCT1-mediated lactate uptake compensates for the inappropriate glutamine supply of PDAC cells subjected to treatment with V9302 or CB839, accounting for resistance against the oxidative-stress-inducing and growth-suppressive effects of these inhibitors. This MCT1-dependent mechanism is another adaptive process to overcome the growth-limiting effects of glutamine-metabolism-inhibitors [63,77] besides alterations in metabolic enzymes such as pyruvate carboxylase [78], which converts pyruvate into oxaloacetate to refuel the TCA. Another important mechanism to overcome glutamine restriction as well as the inhibition of glutamine metabolism by V9302 and CB839 is macropinocytosis [79].

In fact, high levels of macropinocytosis are reported closer to the tumor core, in the nonperipheral areas of xenograft PDAC tumors, compared to peripheral regions. Although an enhanced macropinocytic capacity correlates most significantly with the regional depletion of several nonessential amino acids (NEAA) within PDAC tumors, increased macropinocytosis has been shown to be in response to glutamine scarcity specifically and independent of the absence of any other NEAA like cystine [62]. Indeed, these findings showed that glutamine, particularly, is able to modulate the extent of macropinocytosis in PDAC through the activation of EGFR signaling. These findings correlate with the sensitivity of PDAC cells to glutamine availability and their high reliance on glutamine for metabolic and biosynthetic needs. Another recent study showed that an alternative mechanism to EGFR-dependent macropinocytosis takes place in the PDAC stroma, through AMPK activation by the PDAC stromal cancer-associated fibroblasts (CAFs) in response to glutamine deficiency [80]. CAF macropinocytosis then serves as a source of intracellular amino acids for both tumor cells and CAFs themselves. It could be envisioned that macropinocytosis, to sustain the fitness and function of CAFs, also serves in maintaining their lactate production that thereby enriches the PDAC microenvironment. The ability of cancer cells to utilize MCT1-dependent lactate then spares glutamine and contributes to their survival (as shown in our study). Accordingly, it can be speculated that macropinocytosis is not just an alternative process to retrieve nutrients from the microenvironment, but rather a part of a mechanism orchestrated by glutamine availability, where MCT1 upregulation occurs in concert with increased macropinocytosis under conditions of low glutamine supply. This scenario would correspond to earlier findings that show that macropinocytosis could be triggered under oxidative stress depending on Nrf2 activation [81,82], an activation that was seen in our MCT1-upregulated PDAC cells under glutamine starvation.

Nevertheless, earlier observations have also shown that an enhanced macropinocytic capacity in PDAC cells depends in the first place on its basal level and is largely not affected by glutamine starvation. Indeed, it has been shown that glutamine deprivation strongly enhances macropinocytosis only in PDAC cell lines that display low levels of basal macropinocytosis [62]. It could be speculated based on the data from the two PDAC cell lines used in this study that the time-dependent difference in MCT1 upregulation is also due to differences in their basal levels of macropinocytosis. Its basal levels might be lower in T3M4 cells, triggering a quicker upregulation of MCT1 expression under glutamine starvation conditions. By contrast, in A818-6 cells, an overall higher resistance to glutamine deprivation might be due to a higher basal level of macropinocytosis, thereby leading to a delayed MCT1 upregulation as long as glutamine scarcity persists. Future investigations have to show to what extent MCT1-driven lactate operates as an alternative event to macropinocytosis and/or how these two events act synergistically, e.g., in the context of tumor–stroma interactions with CAFs.

Overall, our findings provide a novel mechanism by which cancer cells, here from PDAC, adapt to metabolic and oxidative stress imposed by glutamine scarcity. Although it is a NEAA and is highly abundant in the body, the availability of glutamine to cancer cells is often limited, particularly in stroma-rich tumors like PDAC. For example, the excessive consumption of glutamine by rapidly growing cancer cells and stromal cells such as CAFs, as well as an impaired perfusion of tumoral areas, may cause cancer cells to suffer from glutamine scarcity. By gaining access to the huge amount of lactate released as a waste product by other cancer and stromal cells, MCT1 helps these glutamine-starved PDAC cells to exploit a metabolite that is excessively available and can be readily as well as variably metabolized [83]. MCT1-driven lactate import results in the efficient protection of PDAC cells from the growth-limiting effect of a reduced glutamine supply, which also manifests after the administration of anticancer drugs targeting glutamine metabolism. This MCT1-dependent mechanism thereby adds to the aggressive and poor prognostic phenotype of PDAC associated with its elevated expression [27,45,84,85,86]. In line with this, previous studies on other tumor entities revealed that oxidative stress adaptation through MCT1-driven lactate uptake manifests not only in primary tumor areas but also during metastases [27,35,43,87,88]. Given these profound effects on cancer malignancy, the targeting of MCT1 becomes a more and more desirable option in anticancer therapy [51,89,90,91].

## 5. Conclusions

Metabolic pressures like glutamine deficiency lead to the emergence of an aggressive and poor prognostic reverse Warburg phenotype in PDAC. As the major fuel of this phenotype, lactate taken up by MCT1 maintains cellular redox homeostasis and thereby cell viability during critical shortages of glutamine supply. This also manifests in resistance against inhibitors of glutamine metabolism, thus limiting their usage in the clinic. Novel therapeutic strategies that target MCT1-expressing reverse Warburg cells will therefore improve metabolic drug therapy responses and thereby the survival rates of PDAC patients.

## Data Availability

All of the data is contained within the article and the Supplementary Materials.

## Supplementary Materials

The following supporting information can be downloaded at: https://www.mdpi.com/article/10.3390/antiox12101818/s1, Figure S1: Specificity control of anti-glutamine staining of PDAC specimens. Figure S2: MCT1 knock-down in T3M4 and A818-6 cells. Figure S3: NRF2 knock-down in T3M4 and A818-6 cells. Figure S4: Lactate protects T3M4 and A818-6 cells from glutamine depletion induced ROS stress. Figure S5: Lactate protects PDAC cells from cell death and cell cycle arrest under glutamine shortage. Figure S6: Figure S7: Lactate protects PDAC cells from V9302 or CB839 induced cell death and cell cycle arrest. Table S1: Lactate protects PDAC cells from cell death and cell cycle arrest under glutamine shortage. Table S2: Lactate protects PDAC cells from cell death and cell cycle arrest under glutamine shortage depending on MCT1. Table S3: Lactate protects PDAC cells from V9302- or CB839-induced cell death and cell cycle arrest Table S4: Lactate protects PDAC cells from V9302- or CB839-induced cell death and cell cycle arrest depending on MCT1.
