# Supplementary material for: Monocarboxylate Transporter-1 (MCT1)-Mediated Lactate Uptake Protects Pancreatic Adenocarcinoma Cells from Oxidative Stress during Glutamine Scarcity Thereby Promoting Resistance against Inhibitors of Glutamine Metabolism
Source: Antioxidants (Basel). 2023 Sep 30;12(10):1818. doi: 10.3390/antiox12101818 (PMC10604597; doi:10.3390/antiox12101818)
Supplement: Supplementary file 1 [file antioxidants-12-01818-s001.zip › antioxidants-2588142-supplementary.pdf]

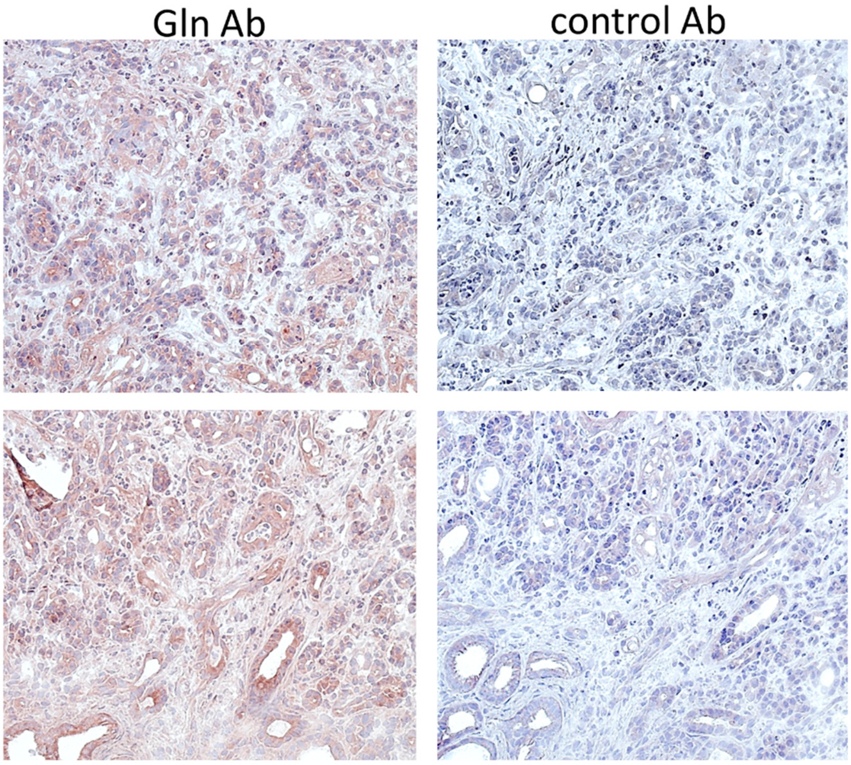

Figure S1. Specificity control of anti-glutamine staining of PDAC specimens. Cryostat sections (6 µm) of snap-frozen specimens from PDAC patients (all classified T3N1M0) were submitted to immunohistochemistry. Following an adapted crosslinking fixation protocol (see materials & methods), sections were probed with a rabbit Glutamine antibody (Abcam, ab9445) or a rabbit control antibody (Abcam, ab172730) at 1:100 dilution in *StainPerfect* antibody diluent and then further developed (see materials & methods). The result with two different PDAC specimens is shown.

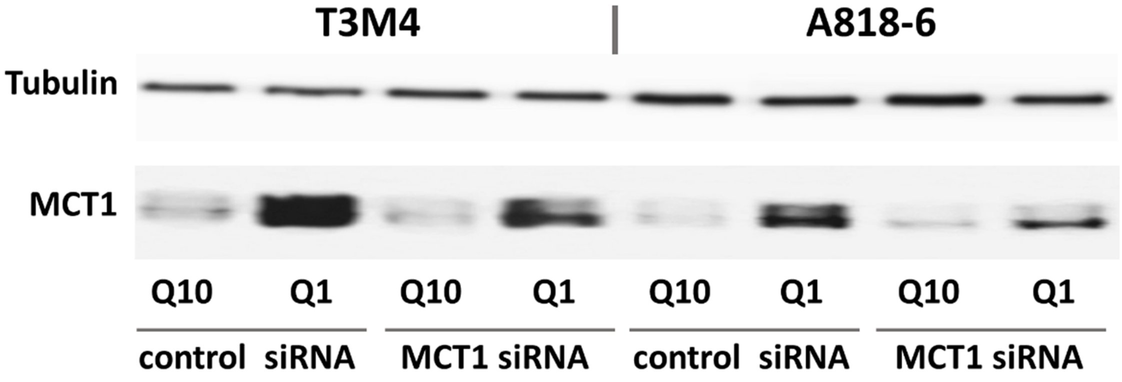

Figure S2. MCT1 knock-down in T3M4 and A818-6 cells. T3M4 and A818-6 cells were transfected with either control siRNA or MCT1 siRNA for 24h and then further cultured under normal glutamine (Q10) or low glutamine supply (Q1) for 48h. A western blot figure from a representative experiment is shown.

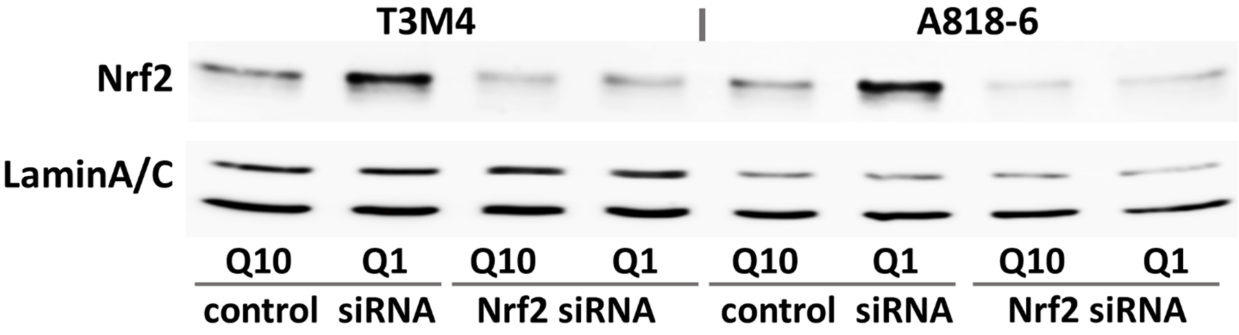

Figure S3. NRF2 knockdown in T3M4 and A818-6 cells. T3M4 and A818-6 cells were transfected with either control siRNA or Nrf2 siRNA for 24h and then further cultured under normal glutamine (Q10) or low glutamine (Q1) supply for 48h. A western blot with nuclear extracts from a representative experiment is shown detecting Nrf2 (Abcam 62352, 1:500 in 5% BSA/TBST) using Lamin A/C (Santa Cruz, 1:1000 in 5% skimmed milk/TBST) as loading control.

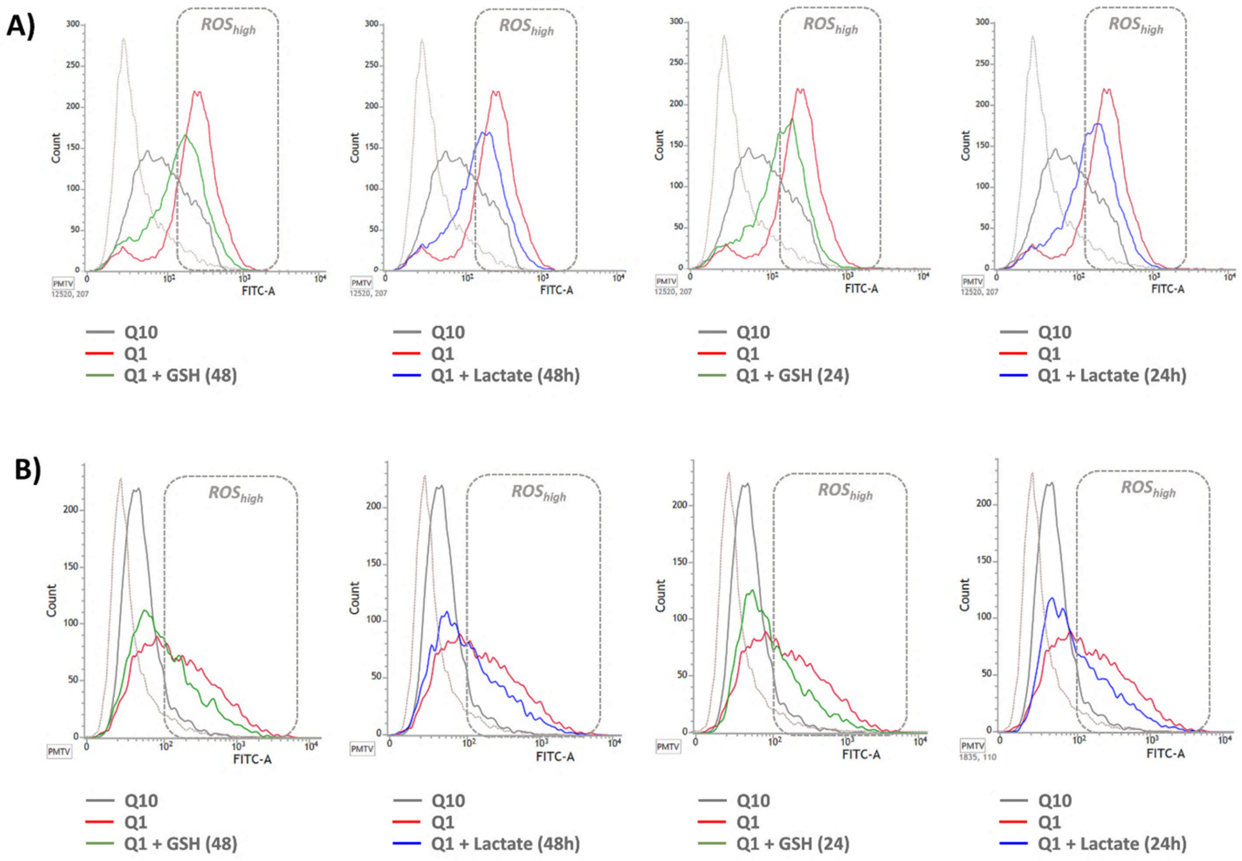

Figure S4. Lactate protects T3M4 and A818-6 cells from glutamine depletion induced ROS stress **A)** T3M4 and **B)** A818-6 cells were cultured under normal glutamine (Q10) or low glutamine (Q1) supply for 48h, either in the absence or presence of 1.5 mM glutathione (GSH) or 20 mM lactate given at the same period or 24h later. Then, cells were DCFDA-stained and analyzed by flow cytometry. Those fluorescence signals >10-fold greater than the background signal with unstained cells (dotted line) were quantified as percentage of highly ROS-stressed (ROS_{high}) cells (see figure 3).

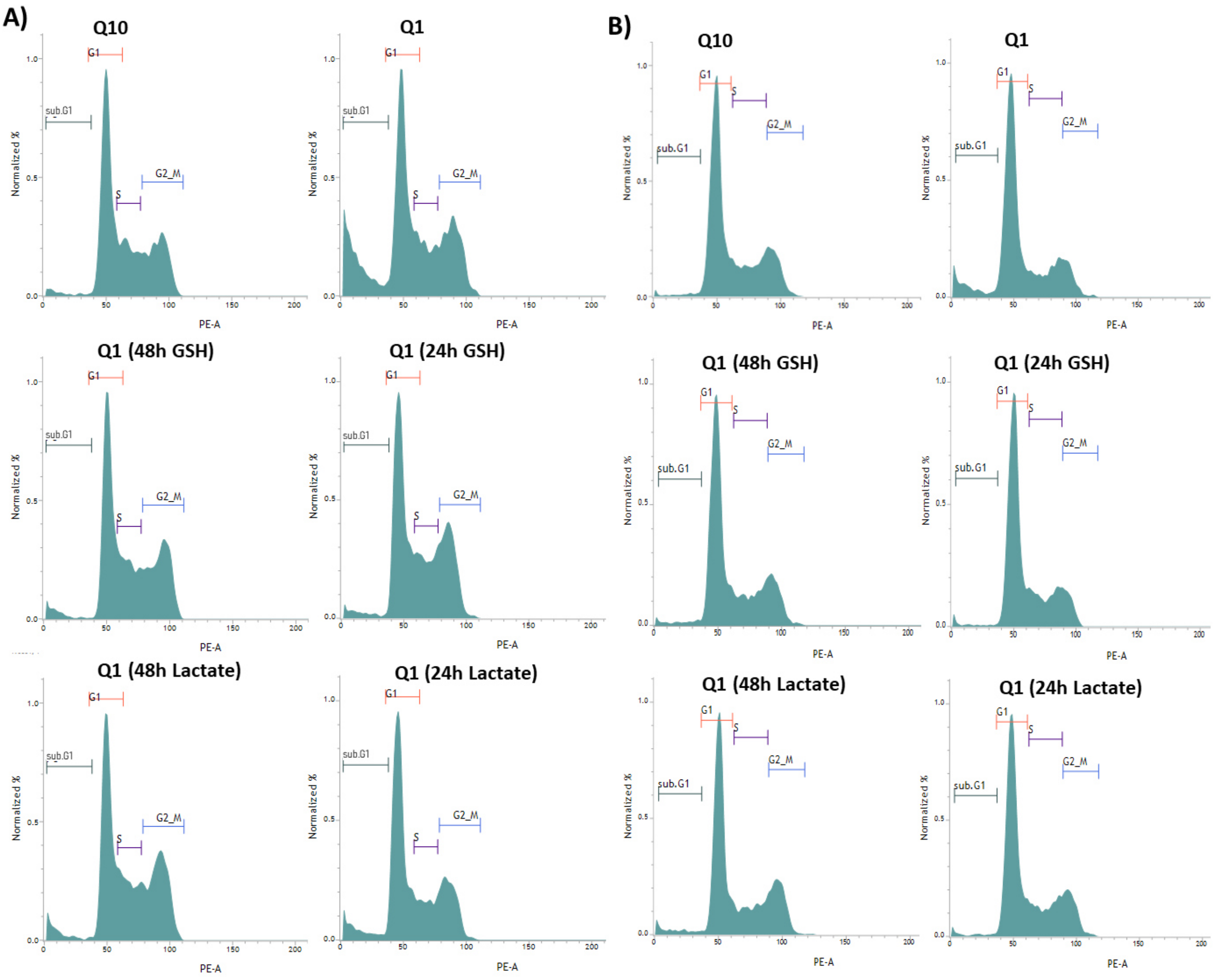

Figure S5. Lactate protects PDAC cells from cell death and cell cycle arrest under glutamine shortage. A) T3M4 and **B)** A818-6 cells cultured with Q10 or Q1 for 48h in the absence or presence of 1.5 mM GSH or 20 mM lactate, added in parallel (48h) or 24h later, were collected and stained with PI and then analyzed by flow cytometry. The subG1 fraction containing apoptotic cells as well as G1, S- and G2/M-cell cycle phase fractions were gated as indicated for quantification (see figure 6).

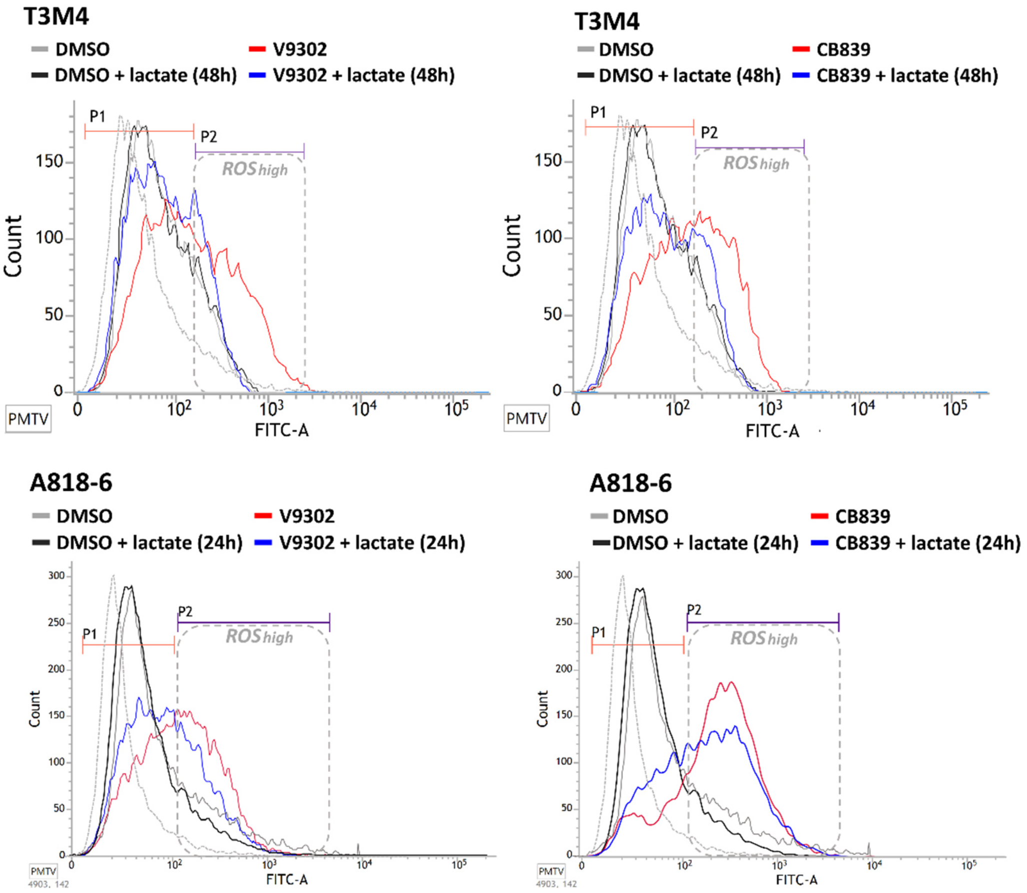

Figure S6. T3M4 and A818-6 cells were treated with V9302 (2 µM), CB839 (10 M) or DMSO for 48h either in the absence or presence of 20 mM lactate as indicated. Then, cells were DCFDA-stained and analyzed by flow cytometry. Those fluorescence signals >10-fold greater than the background signal with unstained cells (dotted line) were quantified as percentage of highly ROS-stressed (ROS_{high}) cells (see figure 8).

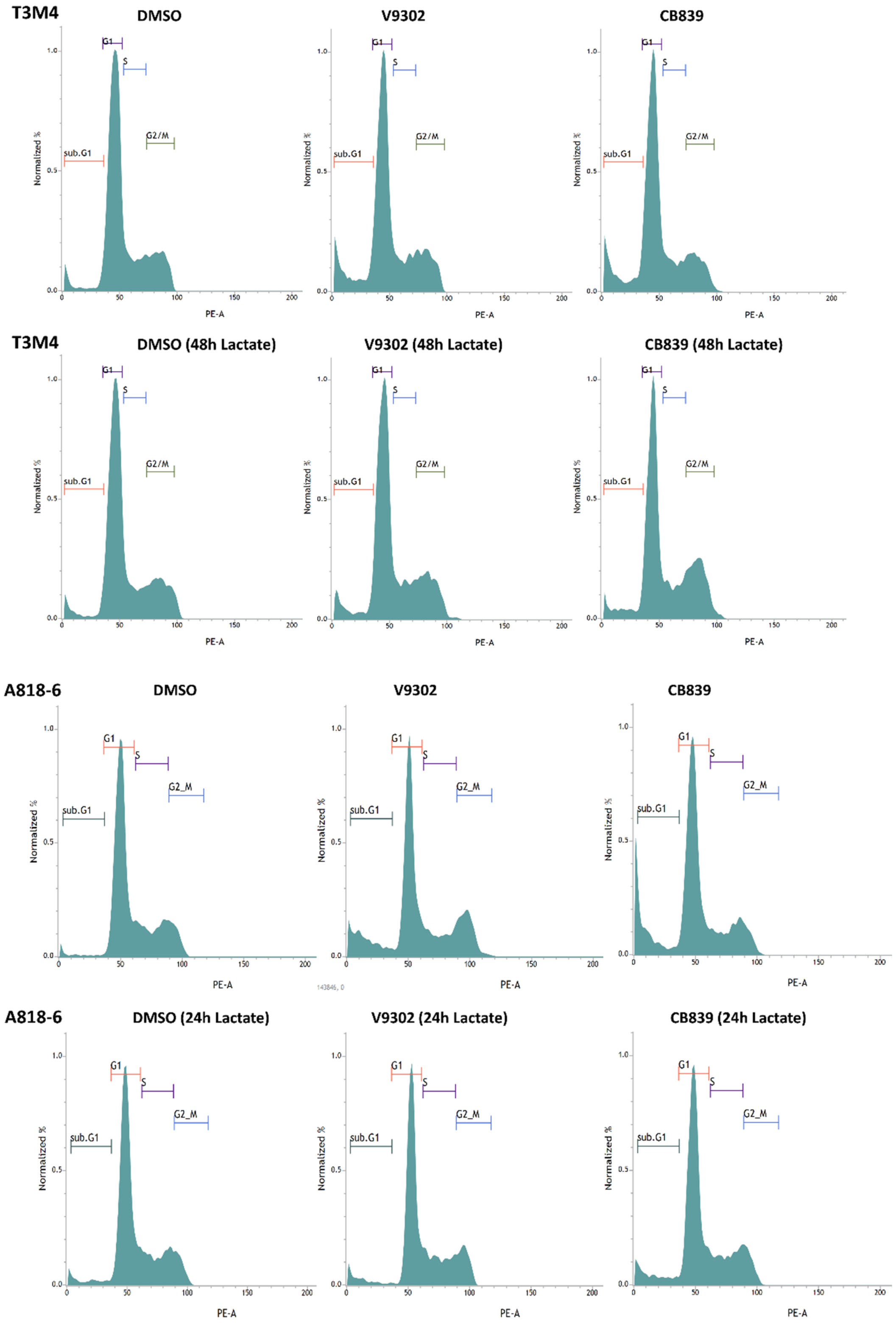

Figure S7. Lactate protects PDAC cells from V9302 or CB839 induced cell death and cell cycle arrest. T3M4 and A818-6 cells were treated with V9302 (2 µM), CB839 (10 µM) or DMSO for 48h either in the absence or presence of 20 mM lactate as indicated. Then, cells were collected, stained with PI and analysed by flow cytometry The subG1 fraction containing apoptotic cells as well as G1, S- and G2/M-cell cycle phase fractions were gated as indicated for quantification (see figure 8).

T3M4 – 24h treatment	G1	S	G2/M
Q10	43,26 ± 4,15	34,21 ± 6,20	22,53 ± 6,91
Q1	53,22 ± 1,98*	28,35 ± 4,12*	18,43 ± 5,12
Q1+24h GSH	45,81 ± 2,85	33,27 ± 5,62	20,92 ± 5,88
Q1+24h Lactate	49,97 ± 2,69$^{\#}$	31,18 ± 5,27	18,86 ± 5,27

T3M4 – 48h treatment	G1	S	G2/M
Q10	57,28 ± 5,17	21,17 ± 3,69	21,55 ± 4,12
Q1	68,15 ± 5,58*	16,23 ± 3,25*	15,62 ± 3,49*
Q1+48h GSH	57,66 ± 3,04$^{\#}$	21,01 ± 3,44$^{\#}$	21,33 ± 3,88$^{\#}$
Q1+48h Lactate	61,55 ± 5,22$^{\#}$	20,10 ± 2,78$^{\#}$	18,35 ± 3,62
Q1+24h GSH	60,64 ± 4,75$^{\#}$	19,82 ± 2,65$^{\#}$	19,54 ± 4,21
Q1+24h Lactate	63,18 ± 5,18	19,14 ± 4,42	17,67 ± 4,24

T3M4 – 72h treatment	G1	S	G2/M
Q10	63,19 ± 7,07	18,80 ± 5,14	18,01 ± 2,84
Q1	68,85 ± 6,21*	15,59 ± 3,25*	15,56 ± 3,49
Q1+72h GSH	65,62 ± 3,94$^{\#}$	16,74 ± 3,16	17,64 ± 3,47
Q1+72h Lactate	63,57 ± 5,25$^{\#}$	19,34 ± 3,51$^{\#}$	17,09 ± 2,69
Q1+48h GSH	65,04 ± 4,16$^{\#}$	17,43 ± 3,08	17,53 ± 2,97
Q1+48h Lactate	65,25 ± 5,29$^{\#}$	18,42 ± 4,34$^{\#}$	16,33 ± 3,26
Q1+24h GSH	66,43 ± 5,86	17,94 ± 3,58	15,63 ± 2,55
Q1+24h Lactate	66,02 ± 4,99	17,91 ± 3,68	16,07 ± 3,53

A818-6 – 24h treatment	G1	S	G2/M
Q10	54,51 ± 4,67	30,14 ± 4,58	15,35 ± 1,77
Q1	59,84 ± 3,84*	27,99 ± 3,65*	12,17 ± 3,23
Q1+24h GSH	55,20 ± 4,02$^{\#}$	31,18 ± 5,84	13,62 ± 2,63
Q1+24h Lactate	58,37 ± 5,79	28,78 ± 5,27	12,85 ± 2,72

A818-6 – 48h treatment	G1	S	G2/M
Q10	50,07 ± 5,03	33,77 ± 4,01	16,16 ± 3,44
Q1	57,78 ± 2,93*	27,29 ± 3,07*	14,93 ± 3,49
Q1+48h GSH	52,29 ± 3,42$^{\#}$	32,62 ± 4,07$^{\#}$	15,09 ± 3,88
Q1+48h Lactate	53,08 ± 3,39	31,74 ± 5,14	15,18 ± 3,62
Q1+24h GSH	51,91 ± 3,22$^{\#}$	31,95 ± 2,65$^{\#}$	16,14 ± 3,08
Q1+24h Lactate	51,35 ± 3,32$^{\#}$	32,82 ± 2,82$^{\#}$	15,83 ± 3,19

A818-6 – 72h treatment	G1	S	G2/M
Q10	55,81 ± 3,75	27,74 ± 4,25	16,45 ± 3,61
Q1	67,59 ± 6,33*	20,22 ± 4,17*	12,19 ± 3,17*
Q1+72h GSH	57,24 ± 3,66$^{\#}$	26,74 ± 4,31$^{\#}$	16,02 ± 3,82
Q1+72h Lactate	58,78 ± 3,83$^{\#}$	25,54 ± 2,97$^{\#}$	15,68 ± 3,00
Q1+48h GSH	59,14 ± 3,27$^{\#}$	25,34 ± 4,81$^{\#}$	15,52 ± 3,24
Q1+48h Lactate	58,25 ± 3,92$^{\#}$	26,78 ± 3,99$^{\#}$	14,98 ± 2,84
Q1+24h GSH	62,06 ± 5,56	23,92 ± 4,11	14,02 ± 2,45
Q1+24h Lactate	57,99 ± 3,52$^{\#}$	27,57 ± 4,05$^{\#}$	14,44 ± 2,78

Table S1: T3M4 and A818-6 cells cultured with Q10 or Q1 for 24h, 48h or 72h in the absence or presence of 1.5 mM GSH or 20 mM lactate added in parallel or later, as indicated, were collected and stained with PI and analyzed by flow cytometry (see Figure 6C). The G1, S and G2/M cell cycle-fractions were quantified. Data are the mean values ± SD from six independent experiments; $^{*}p<0.05$ compared to Q10, $^{\#}p<0.05$ compared to Q1.

T3M4

siRNA	48h treatment	G1	S	G2/M
Control	*Q10*	62.70 ± 4.98	19.12 ± 2.55	18.18 ± 4.12
Control	*Q1*	72.90 ± 6.02*	16.45 ± 2.31*	10.65 ± 2.90*
Control	*Q1+48h Lactate*	65.59 ± 4.86#	19.15 ± 3.00#	15.27 ± 3.25#
Control	*Q1+24h Lactate*	64.74 ± 4.09#	18.76 ± 3.32	16.50 ± 2.77#
MCT1	*Q10*	63.09 ± 3.99	19.37 ± 3.09	18.16 ± 3.85
MCT1	*Q1*	72.03 ± 5.74*	16.83 ± 2.81*	10.42 ± 3.01*
MCT1	*Q1+48h Lactate*	69.68 ± 5.12	16.38 ± 1.95	13.31 ± 2.78
MCT1	*Q1+24h Lactate*	69.87 ± 5.36	16.14 ± 2.23	13.99 ± 3.27

T3M4

siRNA	72h treatment	G1	S	G2/M
Control	*Q10*	65.24 ± 3.55	19.74 ± 2.71	15.02 ± 3.80
Control	*Q1*	69.93 ± 4.69*	17.28 ± 2.60*	12.79 ± 2.13*
Control	*Q1+72h Lactate*	65.64 ± 3.79#	19.53 ± 2.85	14.83 ± 2.42#
Control	*Q1+48h Lactate*	65.31 ± 3.60#	20.03 ± 2.78#	14.66 ± 2.61
Control	*Q1+24h Lactate*	66.62 ± 4.25	18.63 ± 2.34	14.75 ± 2.33
MCT1	*Q10*	67.33 ± 4.52	18.16 ± 2.77	14.51 ± 2.60
MCT1	*Q1*	70.08 ± 4.87	17.01 ± 1.93	12.91 ± 2.24
MCT1	*Q1+72h Lactate*	68.79 ± 4.81	17.93 ± 2.44	13.28 ± 2.09
MCT1	*Q1+48h Lactate*	67.98 ± 4.57	17.49 ± 2.38	14.53 ± 1.92
MCT1	*Q1+24h Lactate*	68.53 ± 4.66	16.70 ± 1.83	14.77 ± 2.42

A818-6

siRNA	48h treatment	G1	S	G2/M
Control	*Q10*	49.47 ± 3.82	30.65 ± 3.01	19.88 ± 2.75
Control	*Q1*	66.57 ± 4.68*	16.80 ± 1.67*	16.63 ± 2.33*
Control	*Q1+48h Lactate*	56.33 ± 3.94#	24.67 ± 2.56#	19.00 ± 2.61#
Control	*Q1+24h Lactate*	54.01 ± 3.79#	27.22 ± 2.65#	18.77 ± 2.50#
MCT1	*Q10*	50.40 ± 4.17	29.24 ± 2.80	20.37 ± 3.00
MCT1	*Q1*	66.73 ± 4.96*	18.56 ± 1.85*	15.37 ± 2.22*
MCT1	*Q1+48h Lactate*	61.54 ± 5.03	20.26 ± 2.18	18.20 ± 3.02
MCT1	*Q1+24h Lactate*	59.87 ± 4.60#	23.76 ± 2.57#	16.37 ± 2.80

A818-6

siRNA	72h treatment	G1	S	G2/M
Control	*Q10*	56,55 ± 3.57	25.02 ± 4,01	18.43 ± 3.21
Control	*Q1*	61.08 ± 4.15*	20.17 ± 3.27*	18.75 ± 3.64
Control	*Q1+72h Lactate*	59.43 ± 3.66	22.44 ± 3.71	18.13 ± 3.10
Control	*Q1+48h Lactate*	57.53 ± 3.72#	23.21 ± 3.52#	19.07 ± 4.05
Control	*Q1+24h Lactate*	54.84 ± 2.99#	23.39 ± 3.16#	19.58 ± 3.23
MCT1	*Q10*	60.30 ± 3.73	22.42 ± 3.47	17.28 ± 3.36
MCT1	*Q1*	61.10 ± 3.47	21.19 ± 2.90	16.93 ± 3.44
MCT1	*Q1+72h Lactate*	60.64 ± 4.13	22.23 ± 3.14	17.13 ± 3.51
MCT1	*Q1+48h Lactate*	60.77 ± 4.00	22.27 ± 3.19	16.96 ± 3.70
MCT1	*Q1+24h Lactate*	59.76 ± 3.54	21.46 ± 2.82	17.44 ± 3.53

Table S2: T3M4 and A818-6 cells pretreated with control or MCT1 siRNA were cultured under Q10 or Q1 for 48h or 72h in the absence or presence of 20 mM lactate added in parallel or later, as indicated. PI-stained cells were quantified for the G1-, S-, and G2/M-cell cycle-fractions (see Figure 6D). Data are the mean values ± SD from four independent experiments;*p<0.05 compared to Q10, #p<0.05 compared to Q1.

T3M4

48h treatment	G1	S	G2/M
DMSO	60.09 ± 2.06	21.56 ± 1.44	18.35 ± 1.75
DMSO+48h Lactate	60.54 ± 2.40	21.46 ± 1.61	18.00 ± 1.78
DMSO+24h Lactate	60.38 ± 1.97	20.46 ± 1.67	19.16 ± 2.06
V9302	79.28 ± 6.23*	12.04 ± 3.14*	8.68 ± 1.86*
V9302+48h lactate	68.79 ± 4.05#	17.33 ± 2.25#	12.88 ± 1.21#
V9302+24h lactate	69.22 ± 4.72#	16.95 ± 2.47#	12.83 ± 1.55#
CB839	68.54 ± 4.23*	16.44 ± 2.66*	15.01 ± 1.93*
CB839+48h lactate	61.34 ± 3.37#	20.76 ± 3.40#	17.90 ± 1.69#
CB839+24h lactate	60.93 ± 3.72#	21.03 ± 3.25#	18.04 ± 1.65#

A818-6

48h treatment	G1	S	G2/M
DMSO	61.62 ± 3.73	29.17 ± 1.95	9.21 ± 1.08
DMSO+48h Lactate	62.00 ± 3.18	28.35 ± 2.01	9.65 ± 1.34
DMSO+24h Lactate	61.83 ± 3.31	28.93 ± 1.82	9.24 ± 1.15
V9302	67.74 ± 3.79*	26.35 ± 2.12*	5.91 ± 0.98*
V9302+48h lactate	64.28 ± 2.89	27.14 ± 1.82	8.58 ± 1.09#
V9302+24h lactate	63.05 ± 3.35#	27.63 ± 1.73	9.31 ± 1.35#
CB839	70.44 ± 3.63*	22.41 ± 1.57*	7.15 ± 1.49*
CB839+48h lactate	64.46 ± 2.55#	25.88 ± 2.74#	9.66 ± 1.25#
CB839+24h lactate	63.56 ± 2.91#	26.73 ± 2.16#	9.71 ± 1.02#

Table S3: T3M4 and A818-6 cells were treated with V9302 (2 µM), CB839 (10 µM) or DMSO for 48h either in the absence or presence of 20 mM lactate for 48h and 24h, respectively. Then, cells were collected, stained with PI and analyzed by flow cytometry (see Figure 8C). Data are the mean values ± SD from four independent experiments; *$p<0.05$ compared to DMSO, #$p<0.05$ compared to V9302 and CB839, respectively, without lactate.

T3M4 siRNA 48h treatment G1 S G2/M

siRNA	48h treatment	G1	S	G2/M
Control	*DMSO*	61.23 ± 5.17	20.09 ± 3.47	18.68 ± 3.53
Control	*DMSO + Lactate (48h)*	61.46 ± 4.75	21.00 ± 3.33	17.54 ± 3.22
Control	*V9302*	78.41 ± 6.76*	13.21 ± 2.19*	8.38 ± 1.84*
Control	*V9303 + Lactate (48h)*	68.21 ± 5.63#	18.29 ± 2.95#	13.50 ± 2.43#
Control	*CB839*	67.92 ± 5.50*	16.55 ± 2.63*	15.52 ± 2.22*
Control	*CB839 + Lactate (48h)*	62.38 ± 4.60#	19.47 ± 3.52#	18.15 ± 3.40#
MCT1	*DMSO*	60.67 ± 3.91	20.38 ± 3.76	18.51 ± 3.31
MCT1	*DMSO + Lactate (48h)*	61.05 ± 4.26	20.17 ± 4.00	18.78 ± 3.64
MCT1	*V9302*	76.52 ± 7.30	14.15 ± 2.52	9.33 ± 2.07
MCT1	*V9303 + Lactate (48h)*	74.28 ± 6.51	15.19 ± 2.49	10.53 ± 2.18
MCT1	*CB839*	69.07 ± 6.11	17.02 ± 2.68	13.91 ± 2.09
MCT1	*CB839 + Lactate (48h)*	68.53 ± 6.26	18.45 ± 3.20	13.02 ± 2.33

A818-6 siRNA 48h treatment G1 S G2/M

siRNA	48h treatment	G1	S	G2/M
Control	*DMSO*	60.83 ± 4.62	29.09 ± 4.76	10.07 ± 2.89
Control	*DMSO + Lactate (24h)*	61.36 ± 5.14	27.42 ± 4.20	11.22 ± 2.95
Control	*V9302*	68.17 ± 6.25*	25.75 ± 4.74*	6.08 ± 1.52*
Control	*V9303 + Lactate (24h)*	62.84 ± 5.20#	27.29 ± 5.11#	9.87 ± 2.84#
Control	*CB839*	69.59 ± 6.49*	22.12 ± 4.57*	8.29 ± 1.97*
Control	*CB839 + Lactate (24h)*	63.12 ± 5.43#	26.04 ± 4.08#	10.84 ± 2.93#
MCT1	*DMSO*	61.29 ± 4.85	27.92 ± 4.98	10.79 ± 2.73
MCT1	*DMSO + Lactate (24h)*	61.91 ± 5.06	27.15 ± 4.72	10.94 ± 2.58
MCT1	*V9302*	69.27 ± 5.70	24.45 ± 3.54	6.28 ± 2.07
MCT1	*V9303 + Lactate (24h)*	68.03 ± 5.28	24.98 ± 4.02	6.99 ± 2.27
MCT1	*CB839*	70.67 ± 6.72	21.63 ± 3.16	7.70 ± 2.38
MCT1	*CB839 + Lactate (24h)*	69.57 ± 5.88	22.28 ± 3.81	8,15 ± 2.33

Table S4: T3M4 and A818-6 cells subject control or MCT1 siRNA transfection for 24h were treated with V9302 (2 µM), CB839 (10 µM) or DMSO for 48h either in the absence (-) or presence of 20 mM lactate for 48h and 24h, respectively. Then, cells were collected, stained with PI and analyzed by flow cytometry (see Figure 8D). Data are the mean values ± SD from four independent experiments; *p<0.05 compared to DMSO, #p<0.05 compared to V9302 and CB839, respectively, without lactate.
